# Diversity, distribution and intrinsic extinction vulnerability of exploited marine bivalves

**DOI:** 10.1038/s41467-023-40053-y

**Published:** 2023-08-15

**Authors:** Shan Huang, Stewart M. Edie, Katie S. Collins, Nicholas M. A. Crouch, Kaustuv Roy, David Jablonski

**Affiliations:** 1 School of Geography, Earth & Environmental Sciences, University of Birmingham, Edgbaston, Birmingham, B15 2TT UK; 2 Senckenberg Biodiversity and Climate Research Center (SBiK-F), Frankfurt (Main), Germany; 3 Department of Paleobiology, National Museum of Natural History, Smithsonian Institution, Washington, DC 20013 USA; 4 The Natural History Museum, London, SW7 5BD UK; 5 Department of the Geophysical Sciences, University of Chicago, Chicago, IL 60637 USA; 6 Department of Ecology, Behavior and Evolution, University of California San Diego, La Jolla, CA 92093-0116 USA; 7 Committee on Evolutionary Biology, University of Chicago, Chicago, IL 60637 USA

**Keywords:** Biodiversity, Conservation biology, Biogeography, Macroecology, Marine biology

## Abstract

Marine bivalves are important components of ecosystems and exploited by humans for food across the world, but the intrinsic vulnerability of exploited bivalve species to global changes is poorly known. Here, we expand the list of shallow-marine bivalves known to be exploited worldwide, with 720 exploited bivalve species added beyond the 81 in the United Nations FAO Production Database, and investigate their diversity, distribution and extinction vulnerability using a metric based on ecological traits and evolutionary history. The added species shift the richness hotspot of exploited species from the northeast Atlantic to the west Pacific, with 55% of bivalve families being exploited, concentrated mostly in two major clades but all major body plans. We find that exploited species tend to be larger in size, occur in shallower waters, and have larger geographic and thermal ranges—the last two traits are known to confer extinction-resistance in marine bivalves. However, exploited bivalve species in certain regions such as the tropical east Atlantic and the temperate northeast and southeast Pacific, are among those with high intrinsic vulnerability and are a large fraction of regional faunal diversity. Our results pinpoint regional faunas and specific taxa of likely concern for management and conservation.

## Abstract (web summary)

Marine bivalves are important components of ecosystems and are exploited for food across the world. This study expands the list of marine bivalves known to be exploited worldwide and then uses a trait-based approach to identify intrinsically vulnerable species and to pinpoint regions with high levels of extinction-prone exploited species, helping to prioritize areas for conservation effort.

## Introduction

Knowledge of the diversity, traits and geographic distribution of marine species harvested for human use is essential for conserving marine biodiversity and predicting its future under global change^1–7^. However, comprehensive lists of exploited species and information about their biology are still lacking for most groups of marine invertebrates^7–9^. Marine bivalves are important components of marine ecosystems^9^ that are increasingly exploited by commercial and artisanal fisheries on a global scale^10–14^. The United Nations Food and Agriculture Organization (FAO) tracks the annual catch of seafood taxa across the world, but data gaps remain for many regions and taxa^15–18^, including bivalves. Even if those data gaps involve taxa with low total catch, consideration of their ecology and vulnerability to over-exploitation is still critical owing to the many ecosystem services provided by bivalves, ranging from ecosystem engineering to their role in many marine food webs. Assessing extinction risk in both taxonomic and biogeographic contexts can strengthen conservation planning and management^19–21^, and is especially desirable when such species loss might affect ecosystem function^22–26^.

Despite the growing catch of marine bivalves^15, 27–30^, their vulnerability to over-exploitation and other anthropogenic pressures^31–34^ is poorly understood^20,35,36^, and direct evaluations of their population sizes and/or conservation status are scarce^37^. For such under-studied taxa, vulnerability models based on biological traits correlated with extinction risk offer a powerful, cost-effective tool for guiding formal risk assessments^38–43^. The rich fossil record of bivalves provides direct correlates of their extinction with biological and biogeographic traits, thus informing predictive models of intrinsic vulnerability for individual taxa under environmental changes^20,37,44^. While some aspects of anthropogenic global change are unprecedented in geologic history, these intrinsic vulnerability models can provide a baseline assessment for identifying geographic regions and phylogenetic clades most susceptible to environmental and climatic instability^20,45^.

Here, we expand the global list of bivalve species harvested for human use (here termed exploited species) using an extensive literature search. We then analyze the phylogenetic distributions, biological traits, and biogeography of these species to assess their intrinsic vulnerabilities to ongoing and future global change. We test for differences between exploited and nonexploited species across several traits previously suggested as predictors of extinction-resistance in marine bivalves and many other clades: body size^46,47^, minimum bathymetry^48,49^, geographic range^50–52^, thermal range^53–55^, and within-clade taxonomic turnover measured in the fossil record, i.e., clade volatility^56,57^. To hypothesize the intrinsic extinction vulnerability of exploited relative to non-exploited species, geographic and thermal range sizes were combined with clade volatility into the PERIL metric (Paleontological Extinction Risk In Lineages), which was defined using known extinction drivers of bivalves and tested on their young fossil record (<5 Ma)^37^.

Following previous studies, we expected that exploited species of bivalves would be larger-bodied^5,58^ and would occur in shallow subtidal or intertidal waters; larger geographic and thermal ranges would also increase their accessibility at regional and global scales. These features may also be correlated with intrinsic extinction resistance, but how those traits are associated with other features, including clade volatility, was unclear. We also expected most exploited species to be tropical, given that bivalve species richness peaks in lower latitudes^59–61^, but the proportion of exploited species might instead relate to areas with long-standing traditions of extracting marine invertebrates. Our trait-based approach identifies intrinsically vulnerable bivalve species, and pinpoints lineages and coastal regions with disproportionally high fractions of extinction-prone exploited species, helping to prioritize areas for further survey and conservation effort.

## Results

### Phylogenetic distribution of exploited species

**Fig. 1:**
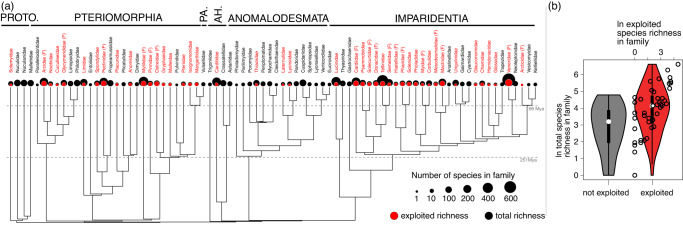
Phylogenetic distribution and species richness of exploited marine bivalve families. Across the 6127 shallow-marine bivalves evaluated here, exploited species cluster phylogenetically from ^107^. Families containing exploited bivalve species (*n* = 44 out of 80, labeled in red and flagged with ‘(F)’ if recorded in the FAO Production Database in panel **a**). These families also tend to be species-rich today (panel **b**), and show a positive correlation between the number of exploited species and total richness (shown by points along the top axis). The violins represent smoothed density of the data, with white dots representing the medians and thick black bars representing the 25–75% quantiles. Clade abbreviations: PROTO. Protobranchia; AH. Archiheterodonta; PA. Palaeoheterodonta. Source data are provided as a Source Data file.

**Fig. 2:**
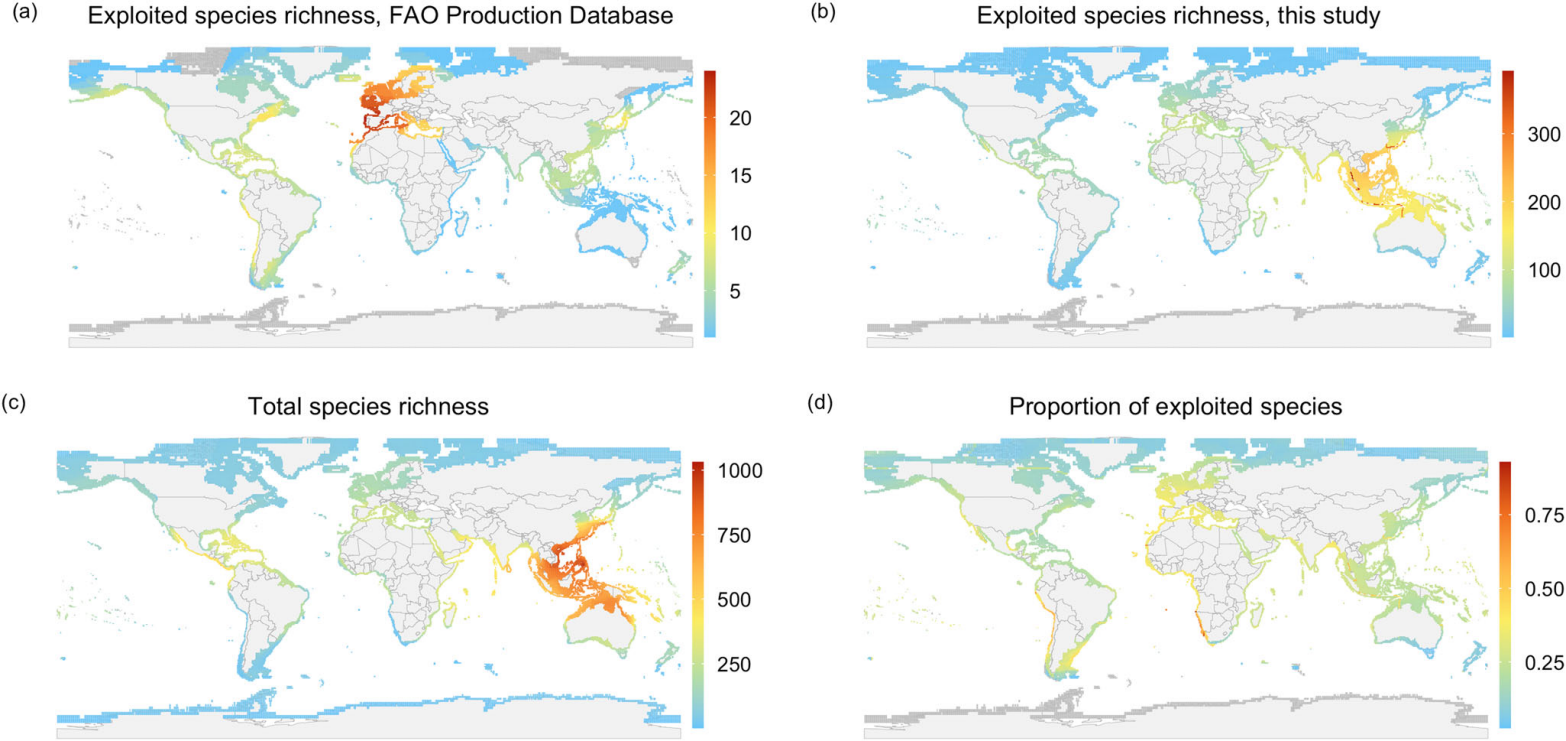
Global distribution of marine bivalves and their exploited species. The geographic distribution of exploited bivalve species (**b**) mirrors the richness distribution of shallow-marine bivalve species today (**c**), but does not coincide with (**a**) the distribution of exploited species in FAO data, or the proportion of exploited species in a 50 × 50 km grid cell. The distributions of exploited species encompasses their natural ranges but do not necessarily reflect where they have been exploited by humans. Source data are provided as a Source Data file.

The literature survey added 720 exploited species (including 490 from FAO Field and Regional Guides) to the 81 species known from FAO Production Dataset (Dataset S1 for sources). The total of 801 exploited species spans 44 of 80 families analyzed here (Fig. 1a), and the 720 species added in this study  alter the spatial distribution of exploited species compared to the FAO Production Dataset (Fig. 2, see below). Exploited species tend to belong to species-rich families, and the number of exploited species in a family increases with total species richness (Fig. 1b, Supplementary Code). The families containing exploited species tend to be weakly clustered on the phylogeny (phylo-D = 0.64, *p* = 0.05) but not following strict Brownian motion (*p* = 0.028; Fig. 1a; Fig. S1). The proportion of exploited species in a family also shows a weak phylogenetic signal (*λ* = 0.33; *K* = 0.63, *p* = 0.05 compared to random distributions), but the absolute number of exploited species does not (*λ* < 0.001; Fig. S1).

### Biological traits of exploited species

**Fig. 3:**
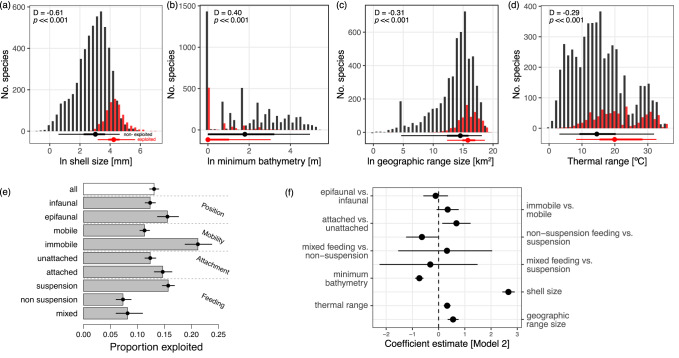
Biological traits of exploited species. Relationships between biological traits and exploitation of 6127 bivalve species. **a–d** Distributions of exploited (red) and non-exploited (black) species across the four continuous traits (shell size, minimum bathymetry, geographic range size, and thermal range). Points and segments below histograms show the median (point), middle 50% (thicker bar), and inner 95% (thinner bar) distributions. The ‘D’ and ‘*p*’ values show results of one-sided Kolmogorov-Smirnov two-sample test. **e** The proportion of exploited species in each functional category (also indicated by points), with lines at the end of the bars indicating the 95% confidence intervals based on a binomial distribution (species counts per functional group in Fig. S2). **f** Estimated effects of traits on exploitation of species from Model 2 with the mean (points) and 95% credible intervals (lines, see full model fit in Supplementary Code). Values above zero indicate a positive correlation between a trait and exploitation, so that the probability of a species being exploited increases with shell size, and wider thermal and geographic ranges, and declines with increasing minimum bathymetry. Magnitude of coefficients reflects relative effect sizes. Differences between functional categories expressed as differences from the model intercept, so that attached species have a higher probability of being exploited than unattached ones. Source data are provided as a Source Data file.

As predicted, exploited species tend to have larger shell sizes than non-exploited species, as well as shallower minimum bathymetric occurrences and larger geographic and thermal ranges (Fig. 3a–d). Shell size has the strongest relative effect on predicting exploitation, followed by approximately equivalent effects of geographic range size, thermal range, and minimum bathymetry (Fig. 3f). Although some differences in exploitation by functional category appear when considering bivalves as a whole (Fig. 3e), after accounting for family-level differences and marginalizing across the continuous traits, only the mode of attachment shows a credible effect on exploitation (Fig. 3f); attached species are more likely to be exploited than unattached ones.

### Intrinsic vulnerability of exploited species

**Fig. 4:**
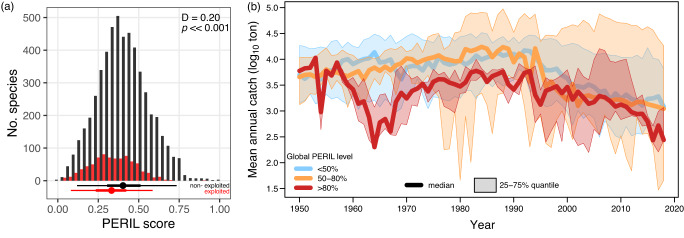
Intrinsic vulnerability of exploited species, and temporal trends in annual catch (where known). **a** Exploited species tend to have lower PERIL scores after accounting for the family membership effect (Model 2), owing to their larger geographic and thermal ranges (Fig. 3c, d; *n* = 801 out of 6127 species). Panel description as for Fig. 3a–d, with the ‘D’ and ‘*p*’ values showing results of one-sided Kolmogorov-Smirnov two-sample test. **b** The 81 bivalve species reported by the FAO Production database through 1950–2018 also include species with PERIL scores above the global median (orange) and even the 80% quantile (red), but species in each category have similar mean annual production to species with lower-PERIL scores (blue). Thick lines represent the median of species annual production for each PERIL category and the shaded polygons show the 25–75% quantiles. Years with zero production were excluded in the calculation and one species, *Arca zebra*, was only reported to be exploited in Bermuda but without any production data. Source data are provided as a Source Data file.

Families with and without exploited species have similar per-capita extinction rates during the Cenozoic ($\hat{q}$) (Table S1). PERIL scores, which express variation along trait axes as hypotheses for overall intrinsic extinction vulnerability, show that exploited species do not follow the same distribution of scores for non-exploited species (Kolmogorov-Smirnov test: D = 0.20, *p* < 0.001). The modeled probability of exploitation decreases with increasing PERIL scores (Fig. 4a, Fig. S3). Exploited species do not have exclusively low PERIL scores (Fig. 4a; top 10 listed in Table 1), with 30% of the species reported in the FAO Production Dataset (24 of 81 species) scoring higher than the global median, of which 7 species (9%) rank above the global 80% quantile (species in each vulnerability category had similar levels of exploitation between 1950–2018, Fig. 4b).

**Table 1:** The 10 most vulnerable exploited bivalve species

Family	Species	Size	MB	GRA	TR	Feeding	Attachment	Mobility	Position	PERIL
Ostreidae	*Crassostrea saidii* Wong & Sigwart in Sigwart et al. 2021	84.9	2	12	2.5	suspension	attached	immobile	epifaunal	0.901
Pectinidae	*Euvola marensis* (Weisbord 1964)	86.9	25	1,868,127	7.2	suspension	unattached	mobile	infaunal*	0.685
Pectinidae	*Leopecten stillmani* (Dijkstra 1998)	84.6	55	44,344	14.5	suspension	unattached	mobile	epifaunal	0.680
Pectinidae	*Euvola laurentii* (Gmelin 1791)	101.0	9	3,218,016	9.1	suspension	unattached	mobile	infaunal*	0.654
Ostreidae	*Saccostrea malabonensis* (Faustino 1932)	58.0	0	463,159	7.1	suspension	attached	immobile	epifaunal	0.643
Veneridae	*Ventricoloidea lyra* (Hanley 1845)	39.9	1	23,439	5.2	suspension	unattached	mobile	infaunal	0.638
**Pectinidae**	***Argopecten purpuratus*** **(Lamarck 1819)**	**157.1**	**0**	**1,384,288**	**12.8**	**suspension**	**unattached**	**mobile**	**epifaunal**	**0.630**
Pectinidae	*Annachlamys kuhnholtzi* (Bernardi 1860)	96.7	4	3,099,169	11.8	suspension	attached	mobile	epifaunal	0.626
Pectinidae	*Equichlamys bifrons* (Lamarck 1819)	132.5	1	1,009,489	14.6	suspension	unattached	mobile	epifaunal	0.617
Pectinidae	*Aequipecten flabellum* (Gmelin 1791)	74.1	1	3,410,268	12.4	suspension	attached	mobile	epifaunal	0.616

Vulnerability was evaluated based on PERIL score and their biological traits, including shell size (Size, mm), minimum bathymetry (MB, m), geographic range area (GRA, km^2^), thermal range (TR, °C) and functional categories. Total ranges for these traits in Fig. 3 and for PERIL in Fig. 4a. The species included in the FAO Production Database is indicated in bold.

*Euvola is a semi-infaunal genus, treated as infaunal for the binary classification used here.

### Biogeography of exploited species

Exploited bivalve species occur along all major coastlines except Antarctica (Fig. 2b, but note that the FAO recognizes potentially exploitable species in these waters^62^, too). Mapped in 50 × 50 km grid cells, the number of exploited species peaks in the tropical west Pacific, the global hotspot of total bivalve richness (Fig. 2b, c). However, less than 25% of the species in this region are documented as exploited (Fig. 2d). The proportion of exploited species tends to be higher along the eastern margins of ocean basins, where the regional faunas are less rich than the west Pacific and most western margins (Fig. 2d, Figs. S4, S5). Comprehensive geographic information of bivalve exploitation is lacking, but these patterns based on their natural occurrences reflect the broadest possible spatial impact that fisheries may have on regional biodiversity.

**Fig. 5:**
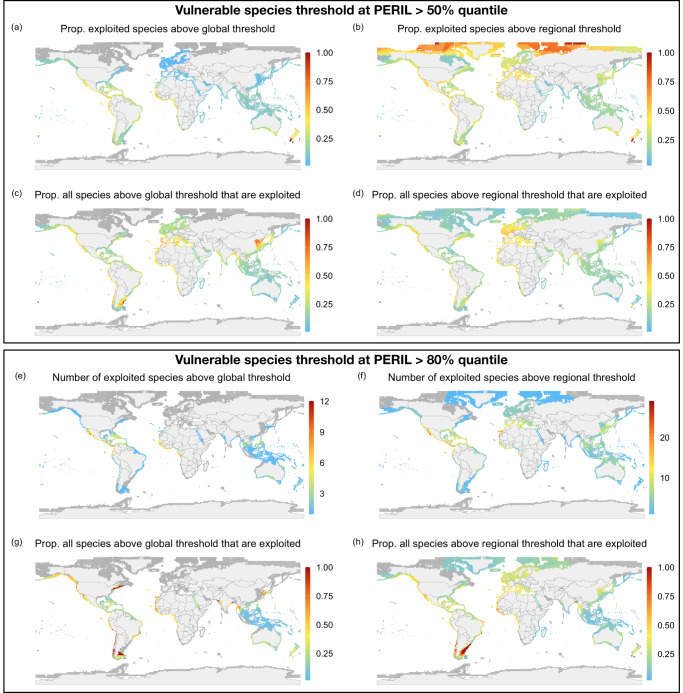
Spatial patterns of intrinsic vulnerability in exploited species. Exploited species occurring in north temperate and polar regions are most prone to extinction (**a, b**) and large proportions of the vulnerable species occurring in these regions are being exploited by humans (**c, d**). Geographic variation was also found in the absolute number (**e, f**) and proportion (**g, h**) of exploited species among the highly vulnerable (top 20%). All maps are in 50*50 km grid cells and the distribution of exploited species do not reflect where the species have been exploited by humans but rather they naturally occur. Vulnerable species are defined by the global (**a, c, e, g**) and region-specific (**b**, **d, f, h**) thresholds. Source data are provided as a Source Data file.

Although exploited species tend to have lower intrinsic vulnerability than non-exploited species at the global scale, exploited species can have relatively high intrinsic vulnerability within certain regions. Compared to the tropics, exploited species found along temperate and north polar coastlines are a larger proportion of the intrinsically vulnerable species (using the median PERIL scores, i.e. 50% quantile; Fig. 5a, b, Figs. S7, S8), and these regions also have higher proportions of vulnerable species being exploited overall (Fig. 5c, d). Greater proportions of highly vulnerable, exploited species are in temperate regions (Fig. 5g, h, Fig. S9), in terms of raw numbers, but the most vulnerable exploited bivalves (PERIL score higher than 80% species globally or regionally) are concentrated on both coasts of Central America and the west coast of Africa; the pattern remains at both lower and higher threshold values, see Figs. S10, S11).

## Discussion

### Expanded global inventory

Humans exploit many more marine bivalve species than suggested by the current phylogenetic and geographic scope of the FAO Production Database. Most information in the primary literature on exploited species is limited to simple characterizations that they are harvested and/or consumed by humans, but these data now expand the FAO production inventory from 81 to 801 bivalve species, and from 17 to 44 families—an expansion that is probably still incomplete. Most added diversity is from the heavily exploited major groups covered by the FAO: the Imparidentia and Pteriomorphia (Fig. 1a). Still, we recovered exploited species from unexpected phylogenetic positions: among the mostly small-bodied and deep-sea Protobranchia and Anomalodesmata, and among the mostly small-bodied Archiheterodonta (Fig. 1a). Thus, for this dataset, humans exploit ~13% of shallow-marine bivalve species richness, half of its family-level phylogenetic diversity, and virtually all of its major body plans.

Despite reporting catch data from 58 different countries or regions (as recognized by the United Nations), the geographic coverage of the FAO is spatially biased. Between 1950–2018, catch from only three species was reported in southeast Asia and none from mainland China—two biodiverse regions (Fig. 2c; Fig. S12) with intensive use of bivalves as food sources^11,63,64^, where other exploited species in the FAO dataset are known to occur naturally (Fig. 2b). By comparison, less biodiverse regions such as southern Europe and the Americas reported more than 20 exploited species in recent years (Fig. S12). The 720 exploited species added in this expanded dataset mostly come from the tropical west Pacific fauna (270 species), the Indian Ocean (247 species) and the temperate west Pacific fauna (176 species), where the FAO has fewer data (compare Fig. 2a,b). Many of those species likely represent local or regional artisanal fisheries that fall beyond the remit of the FAO^15,16,18,65,66^. Monitoring these populations remains important because their local extinction by overexploitation can have large impacts on ecosystem health and regional economies^25,67,68^. Further, surveying the extent of exploitation, and its impact on exploited populations will provide crucial information for sustainable management.

### Intrinsically vulnerable clades and traits

Whether a bivalve family contains exploited species is phylogenetically structured, suggesting that further exploitation is likely to occur within those families and larger clades, such as the Pteriomorphia, a higher taxon rich in exploited species in both absolute and relative terms (e.g. compared to the more diverse Imparidentia). The number of exploited species within their respective families does not show a phylogenetic signal, presumably owing to factors such as total species richness and family-level biogeography. However, improved phylogenetic resolution in future studies may yield finer-scale phylogenetic signals within families, e.g. related to body size or bathymetry.

If overexploitation drives the decline or extinction of large-bodied and widespread species, exploitation will likely shift along phylogenetic lines towards confamilial species^2^, which will be smaller-bodied and narrower-ranging and thus more extinction-prone^37,46,47,52,69^. Such shifts towards the more vulnerable pool of exploited species have been documented in terrestrial vertebrates^1^ and edible plants^4^. In bivalves, scallops and oysters may be on this path, as they have among the highest proportions of exploited species with high intrinsic vulnerability (i.e. in the top 20% PERIL scores: 26 of 62 exploited Pectinidae species and 9 of 38 exploited Ostreidae; see also Table 1); the two families thus appear to be clear priorities for further assessment and monitoring. For example, the pectinid *Hinnites corallinus* has recently been flagged as “potentially exploitable”^70^ but is in the top 2% of bivalve PERIL scores and so is highly vulnerable. Beyond their use as a human food source, the Ostreidae and Pectinidae are particularly important components of natural food webs and include ecosystem engineers that clean the water column by filtering suspended sediment and microbes^9,71–73^ and create hard substrata including “reefs” that reduce shoreline erosion and support higher diversity of commercially important fishes^74^. Extinctions or severe declines in the abundance of such species and other exploited species, including tellinids, whose siphons are a major food source for exploited demersal fishes such as flounder^26^, could have cascading bottom-up effects on marine biodiversity^3,67,75,76^. Fossil data indicate that scallops are especially extinction-prone, with the second highest Cenozoic extinction rate among bivalve families^37^, and this may amplify their intrinsic vulnerability to exploitation and environmental change today ^e.g.^^77,78^.

Perhaps paradoxically, traits associated with the exploitation in marine species also increase their extinction resistance. Exploited bivalve species tend to have large thermal ranges (and overall lower PERIL scores), suggesting a potential for physiological tolerance to changing temperature and thus lower extinction risk^59,69,79^. Exploited bivalve species also tend to have larger body sizes, which is not surprising as larger bodies provide a higher nutritional return per specimen^5^. Large body size also relates to higher fecundity, thus promoting replenishment of exploited populations and extinction-resistant, broad geographic ranges^80–82^. However, some exploited species do have narrow geographic ranges and are in families with histories of high extinction rates, and thus are among the most intrinsically vulnerable bivalves (Fig. 3, Table 1). More importantly, even the most fecund and widespread bivalves—otherwise well suited to cope with global environmental and climatic changes^49,83^ and even artisanal harvest^84^—cannot withstand intense industrial exploitation^85,86^. For example, the  collapse of large-bodied, intertidal and shallow subtidal Eastern Oysters before the 20th-Century^85,87,88^ shows that exploitation alone can quickly reduce biological and ecological diversity in regional faunas.

### Biogeography of vulnerability

Exploited bivalve species and their intrinsic vulnerabilities are also distributed unevenly across geographic regions, and our analyses provide a global picture of where further monitoring and conservation efforts would be especially worthwhile. Exploited species comprise a greater fraction of regional diversity outside of the global marine biodiversity hotspot, suggesting a disproportionate impact on certain regional faunas. The high proportion of exploited species on the southeastern coastlines of the Atlantic and Pacific coincide with high-intensity upwelling^89^, which elevate nutrient availability and, at least in some bivalve clades, body sizes^90,91^ (and thus their exploitability, Fig. 3a); however, we find inconsistent differences in the body sizes of bivalves in these regions compared to those with lower-intensity upwelling (Fig. S6). A nonexclusive alternative is that the high proportion of exploited species might result from a drop in total diversity relative to neighboring regions^91,92^, in combination with the tendency of exploited species to be widespread, rather than an increase in numbers of exploited species (cf. Figure 2c, d). Exploited species with high intrinsic vulnerability do not entirely coincide with these regions of high proportional exploitation. The two areas overlap along the temperate southeast Pacific (cf. Figure 2d, Fig. 5h), but intrinsically vulnerable species also reach high numbers along the northeast Pacific, tropical east Atlantic and tropical to southwest Atlantic (Fig. 5h). The uneven distribution of intrinsic vulnerabilities may derive from the narrow thermal and geographic ranges imposed by steep climatic gradients and narrow coastlines, as seen along the entire east Pacific and parts of the tropical east Atlantic. Thus, certain regions lacking high proportional exploitation harbor exploited species having high intrinsic vulnerability, representing additional areas of potential concern and thus candidates for targeted studies (i.e. the southwest Atlantic and tropical east Atlantic Fig. 5h).

### From vulnerability estimation to risk assessment

Our analyses serve as a first-order assessment of the relative intrinsic vulnerability of species globally and within regions (i.e. their sensitivity to perturbation^93^), which should provide a basis for further assessment of individual species and regional biotas in the future^43^. We emphasize that our results represent *relative* measures of vulnerability that should be used as a starting point for investigating the *absolute* vulnerabilities of these species and their intersection with other anthropogenic stresses^94^. The IUCN Red List provides a framework for assessing the absolute extinction risks of species, but fewer than 50 shallow marine bivalve species have been evaluated so far^95,96^. The PERIL framework can help to prioritize species where formal risk assessments^37,39,45,97^ would be beneficial.

The low PERIL value of a species should not promote complacency, let alone intensification of exploitation, as continued anthropogenic impacts can undermine the natural intrinsic extinction resistance of species. Biodiversity management increasingly takes into account the direct impact of human activity^35,36,39,65,97,98^—a strong component of extinction risk—and applying a trait-based framework to marine bivalves highlights geographic and phylogenetic areas to concentrate future, more detailed studies of human impact. As a start, the exploited species displaying relatively high vulnerability (e.g. Table 1) are primary targets for quantitative analyses of catch metrics and stock assessment. In fact, nearly a third of the marine bivalve species listed by the FAO, which primarily documents sustained and higher levels of exploitation, have higher-than-average PERIL scores, i.e. they are intrinsically more vulnerable to extinction. If bivalves are poised to be a strong component of the Blue Foods movement^64^, providing sustainable nutrition for the world’s population, our analyses show that stronger assessments of their extinction vulnerability will be critical, especially in light of more general calls (e.g. IPBES 2019^7^) to rapidly expand our identification of nature’s contributions to people and the direct threats of anthropogenic pressures to the persistence of species and their ecosystem services. Extensions of our analyses should include, for example, changes to intrinsic vulnerability when geographic ranges of species expand due to human introductions into new areas, and should also investigate why natural populations often fail to recover when exploitation is reduced or stopped: for example, are they entirely driven by crossing demographic thresholds driven by human-stressors, such as habitat conversion and pollution^84,86,99–102^, and/or other ecological interactions such as changes in food web structure^3,75,76^?

### Summary

Using a database of exploited bivalve species that is larger by nearly an order of magnitude than the widely used FAO Production Database, we highlight the utility of an integrative, trait-based approach to understanding the effects of human exploitation and extinction risk in taxa that have been understudied, yet represent an important component of global marine biodiversity. The PERIL framework used here draws on a simple but informative set of variables to compare extinction vulnerability among species in a globally exploited system, making it a useful tool for guiding future surveys and monitoring efforts. We found that bivalve species exploited by humans tend to have intrinsic characteristics that generally promote resilience to environmental changes (e.g. larger geographic and thermal ranges). Nonetheless, our analyses identified a disproportionate number of exploited species with high intrinsic vulnerability to extinction in several clades, most notably in Pteriomorphia, and geographic regions, most notably the tropical east Atlantic and southwest Atlantic and southeast Pacific. Those species and clades urgently need further assessments of: a) the current status of population sizes and health, b) the nature and impact of human exploitation, and c) future vulnerabilities owing to accelerating regional warming and ocean acidification. Comprehensive investigations of the general mechanisms and consequences of bivalve extinction in a biogeographic framework can help develop effective management of marine resources and biodiversity. Thus, PERIL scores can be an element in the development of new strategies in the sea for “catching and cultivating wisely”^65^.

## Methods

### Bivalve exploitation, biogeography, traits, and fossils

We compiled a dataset of exploited intertidal and shelf-depth (<200 meters) marine bivalve species from the FAO Global Statistical Collections and an extensive literature survey (see the workflow for compilation in the Supplementary Methods and references in Dataset S1). The FAO list of exploited species was based on the production data for Bivalvia from the Global Capture Production dataset (annual production during 1950 to 2018 by country, with Bivalvia listed as an “order”: https://www.fao.org/fishery/en/statistics, accessed on August 6th, 2020). From the primary literature, we included all marine bivalve species that have been reported as a food source. Most species are simply listed as being consumed by humans, without further information on catch or habitat (e.g. *Divalinga quadrisulcata* listed as “edible” in Huber^103^), but some are investigated in detail (e.g. *Anadara tuberculosa*, whose fishery was studied in ref. ^104^). Species names were standardized using a global bivalve diversity database^61,105^ with updates primarily based on^103,106^.

Of the 100 currently recognized marine bivalve families in MolluscaBase (as of Nov. 1, 2021), 80 families were analyzed here because they could be placed in the phylogeny^107^ (further details in Supplementary Methods). Global marine bivalve occurrence data on species occurring from the intertidal zone to the 200 m isobath were analyzed using a global bivalve diversity database^61,105^ with updates primarily following Huber^103^ and von Cosel & Gofas^61,106^ (77,190 point occurrences across 7,410 localities and 6,127 species). These natural geographic occurrences of bivalve species are unrelated to whether or not the species is fished at a given locality. For each species, a convex hull of all point occurrences were intersected with an equal-area grid of the continental shelf (50 km × 50 km; ~0.5° latitude-longitude at the equator in a Lambert cylindrical equal-area projection; Dataset S2). To minimize false occurrences derived from misidentifications and misprints of locality names or coordinates, the two-dimensional species’ distributions were critically checked against primary literature sources, synoptic regional global revisions, and global datasets^103,106,108,109^. The finer details of the species’ geographic ranges are subject to further revision given continued discovery and taxonomic revision, but this dataset is expected to be robust to regional-scale sampling biases^60^.

Species traits were compiled from multiple sources, as outlined below and in Dataset S1. Following Collins et al.^37^, a geographic range size (km^2^) was calculated as the area of the convex hull of each species’ point occurrences, and the thermal range (C°) was the range of satellite-derived sea surface temperatures from MARSPEC^110^ encompassed by the species’ grid cell occurrences (MARSPEC derives climate data from both NOAA’s World Ocean Atlas^111^ and NASA’s Ocean Color Web^112^). A large geographic range calculated in this way affords a species a greater chance of surviving a local or regional perturbation, providing the reach of that event is smaller than the range of the species; this overall range extent relative to potential perturbations effectively views extinction risk in terms of spatial autocorrelation of environmental stresses (see ref. ^37^). Species known only from a single point occurrence (580 species) were operationally assigned a range size of 10x10 km (100 km^2^), but some single-occurrence species retained the area of their known limited range, e.g. from a single estuary^37^. Species body size (mm) was measured as the geometric mean of the shell length (from the anterior to posterior margin) and height (from the dorsal to ventral margin) of the largest specimen found in the literature^113^ (with data sourced from Berke et al.^114^ and updated from Huber^103^). The largest specimen represents the potential size for organisms with indeterminate growth such as marine bivalves, reptiles and amphibians and is a typical species body size measure for comparative studies on these taxa^114,115^. The minimum bathymetry (m) was based on the reported bathymetric range in the literature (sourced from^103^). For 191 species lacking precise bathymetric data, we used an estimated minimum bathymetry of 5 m for “subtidal” or “sublittoral” species and 10 m for species in “shallow water”; analyses excluding these species produced consistent results (Supplementary Code). The feeding type, living position, substratum attachment, and mobility of species were from^61^. The intrinsic turnover rate of species was characterized by the per-taxon extinction rate of genera within their family through the Cenozoic (data from^37^); these rates were determined using the first and last stratigraphic occurrences of bivalve genera, which are considered to be more robustly sampled than species but share similar macroevolutionary properties^56,57,116^ (further discussion in ref. ^37^). All variables have 100% coverage in our dataset except minimum bathymetry (95.7% data coverage, missing for 265 non-exploited plus 2 exploited species).

### Phylogenetic distribution of exploited species

The distribution of exploited taxa across the time-scaled family-level bivalve phylogeny^107^ was analyzed in two ways: first, as the presence or absence of any exploited species in a family using phylo-D (for a binary trait)^117^, and second as the proportion of exploited species in a family (i.e. a continuous variable) using Pagel’s *λ* (for a continuous trait)^118,119^.

To assess whether exploited species occur in more diverse families, we assessed exploitation in relation to the total species richness in a family using a Bayesian, hierarchical regression model (specified in Supplementary Code): exploitation ~ richness + (1 | family phylogeny). The exploitation term was modeled separately as either a binary indicator of the family containing exploited species or as the number of exploited species in a family. Potential phylogenetic effects were accounted for by modeling the variance-covariance matrix of the family phylogeny^120–122^.

### Biological traits of exploited species

Using Bayesian hierarchical regression models (specified in Supplementary Code), we compared four continuous biological traits between exploited and non-exploited bivalve species (geographic range size, thermal range, body size, and minimum bathymetry) and four categorical functional traits (position, mobility, attachment, feeding). Potential phylogenetic effects were accounted for by modeling two group-level (random) effects: (a) family membership (as “family” below) and (b) the variance-covariance matrix of a time-scaled family phylogeny (as “family phylogeny” below):

- Model 1: exploitation ~ trait_1_ + … + trait_n_;
- Model 2: exploitation ~ trait_1_ + … + trait_n_ + (1|family);
- Model 3: exploitation ~ trait_1_ + … + trait_n_ + (1|family phylogeny);

where “trait_n_” is one of the eight species traits defined above. Continuous trait values were z-standardized to better compare their effect sizes. Each model was run for five chains of 15000 iterations, discarding the first 5000 iterations as burnin, resulting in 50000 posterior samples. In all models, we used the default priors and sampling algorithms (Hamiltonian MCMC) provided in the package ‘brms’^123,124^. All credible intervals of fit parameters were defined as the 2.5–97.5% quantiles of the posterior samples. Expected log pointwise predictive density (ELPD) from leave-one-out cross-validations was used to compare the fits across all three models, and leave-one-group-out cross-validation was used to compare the fits of models 2 and 3 given the different structure of their random effects (following^125^).

For all the species-level traits, Model 2 and 3 performed similarly (ΔELPD < 2), both significantly better than Model 1 (ΔELPD > 10; see Supplementary Code for details), and so results were interpreted with respect to Model 2 where family membership was the only group-level effect. Thus, exploitation covaries with species’ traits among families, but the phylogenetic relationships of those families does not predict additional covariance, possibly owing to the similarity of species’ traits across families separated by more than 100 Myr of evolutionary time^107^ (Fig. 1a).

### Intrinsic vulnerability of exploited species

The relationship between exploitation and historical extinction $\hat{q}$ was assessed at the family level, accounting for phylogeny: exploitation ~ $\hat{q}$ + (1 | family phylogeny) (Supplementary Code, similar to models of species richness above). The exploitation term was modeled as a binary indicator of whether a family contains exploited species.

We combined geographic range size and thermal ranges with family extinction history in a composite metric, PERIL^37^, to assess the intrinsic vulnerability of exploited species to ongoing and future environmental changes:

1 $${{{{{\rm{PERIL}}}}}}=\hat{q}+\frac{1}{{{{{\mathrm{ln}}}}}\,r}+\frac{1}{T},$$

where $\hat{q}$ is the genus-level per-capita extinction rate of each target species’ family through the Cenozoic^56,57,126^, and *T* is the realized thermal range within the species’ geographic range *r*. Geographic and thermal ranges are inverted so that larger ranges are associated with lower extinction risk (the metric was validated via survivorship analyses in the Pliocene fossil record^37^). Both the geographic and thermal ranges are informative because widespread species are not necessarily eurythermal (Fig. S13); many widespread species track a narrow range of sea-surface temperature across the large tropical or polar areas^127^. To equalize each variable’s contribution to the PERIL score, $\hat{q}$,*r*, and *T* are rescaled from their respective ranges across the dataset to between 0 and 1. Thus, the PERIL score reflects a relative estimate of intrinsic vulnerability among analyzed species^37^. Data for both $\hat{q}$ and PERIL scores are included in the species trait dataset (Dataset S1).

We compared the PERIL scores of exploited and non-exploited species using the same modeling framework as for the continuous species traits above (Bayesian, phylogenetic hierarchical regression models, see Supplementary Code). Model comparisons indicated limited effect of family phylogeny, and results were interpreted with respect to Model 2 where family membership was the only group-level effect.

### Biogeography of exploited species

Spatial patterns of exploited and non-exploited species richness, and their intrinsic vulnerability (based on the PERIL scores), were quantified using their occurrences on the global 50 × 50 km grid intersected with the continental shelf. These geographic ranges reflect the known, natural extents of species, and do not include human-introduced occurrences via aquaculture or unintentional introductions (following the protocol for other studies of model systems in macroecology, e.g. birds and mammals^128,129^). Natural geographic ranges are likely to be the reservoirs for genetic variation within the species and so are of particular interest for conservation. Because initial analyses showed higher proportions of exploited species along east margins of major ocean basins, and that body size was the best predictor of species exploitation, we also tested for differences in the distributions of body sizes of species among regions (defined by the intersection of major climatic zones and coastlines in Fig. S4, following^130–132^) using Wilcoxon rank-sum tests.

Spatial variation in the extinction risk of exploited species was analyzed at two scales. We calculated both the number and proportion of exploited species in a grid cell with PERIL scores above the global median (globally vulnerable species) or above the median within a region, i.e. the intersection of coastlines with polar, temperate, and tropical climate zones (regionally vulnerable species). We further identified highly vulnerable species as those with PERIL scores in the top 20% globally or regionally (see additional cutoffs shown in Figs. S10, S11). Using thresholds to define species with high risk of extinction is analogous to the large body of literature studying “threatened species” in mammals and amphibians^133–135^, for which direct evidence on threats is more readily available for global assessments (e.g. by the IUCN).

All data were processed and analyzed in R 4.2.2^136^ with the packages ‘tidyverse’^137^, ‘Hmisc’^138^ and ‘ggtree’^139–141^ for dataset visualization and transformation, ‘sf’^142^ for spatial analyses, ‘ape’^143^, ‘phytools’^144^ and ‘caper’^145^ for phylogenetic analyses, and ‘brms’^123,124^ and ‘loo’^146^ for Bayesian multilevel regression analyses and model comparison.

### Reporting summary

Further information on research design is available in the Nature Portfolio Reporting Summary linked to this article.

## Supplementary information

Supplementary Information

Description of Additional Supplementary Files

Supplementary Dataset 1

Supplementary Dataset 2

Supplementary Code

Reporting Summary

## Data Availability

All data used in this study are provided in Supplementary Information, Supplementary Data 1, 2. Source data for all figures are provided with the paper. The posterior samples from Bayesian models generated in this study and the shapefile of the global equal-area grids are deposited at Figshare^147^ (10.6084/m9.figshare.22696279). Source data are provided with this paper.

## Source data

Source Data

## Code availability

All analytical code is provided in Supplementary Code. The code for producing all figures and tables is deposited at Figshare^147^ (10.6084/m9.figshare.22696279).
