# Supplementary material for: Diversity, distribution and intrinsic extinction vulnerability of exploited marine bivalves
Source: Nat Commun. 2023 Aug 15;14:4639. doi: 10.1038/s41467-023-40053-y (PMC10427664; doi:10.1038/s41467-023-40053-y)
Supplement: Supplementary file 1 — Supplementary Information [file 41467_2023_40053_MOESM1_ESM.pdf]

Supplementary information for:

Diversity, distribution and intrinsic extinction vulnerability of exploited marine bivalves

Shan Huang[1, 2], Stewart M. Edie[3], Katie S. Collins[4], Nicholas M. A. Crouch[5], Kaustuv Roy[6], David Jablonski[5, 7]
(These authors contributed equally: Shan Huang, Stewart M. Edie)

[1] School of Geography, Earth & Environmental Sciences, University of Birmingham, Edgbaston, Birmingham, B15 2TT, UK
[2] Senckenberg Biodiversity and Climate Research Center (SBiK-F), 60325, Frankfurt (Main), Germany
[3] Department of Paleobiology, National Museum of Natural History, Smithsonian Institution, Washington, DC, 20013, U.S.A.
[4] The Natural History Museum, London, SW7 5BD, United Kingdom
[5] Department of the Geophysical Sciences, University of Chicago, Chicago, IL, 60637, U.S.A.
[6] Department of Ecology, Behavior and Evolution, University of California San Diego, La Jolla, CA 92093-0116, U.S.A.
[7] Committee on Evolutionary Biology, University of Chicago, Chicago, IL 60637, U.S.A.
*Corresponding author: s.huang.7@bham.ac.uk

Table of contents

Supplementary Methods 2

Supplementary Table 3

Supplementary Figures 4

Supplementary References 17

Supplementary Methods

Workflow for compiling exploited species dataset.

1. The FAO Global Capture Production dataset (annual production during 1950 to 2018 by country, with Bivalvia listed as an "order": https://www.fao.org/fishery/en/statistics, accessed on August 6th, 2020) was downloaded as the initial set of exploited species.
2. Next, two FAO Publication Topics were exhaustively searched for records of bivalve species outside of those known from the Global Capture Production dataset: Topic 18083 Regional Guides for Species Identification for Fisheries Purposes; Topic 18079 Fish Finder Field Guides; Topic 18073 Species Catalogues. The 17 publications containing exploited species outside the Global Capture Production dataset are referenced in Dataset S1 [sheet: exploited_references], flagged as 'FAO_source' = 'yes'. Duplicated species records were not recorded.
3. Huber 2015 (his supplementary dataset provided on CD-ROM with book) was then searched for additional references to exploited species using the keywords:
    - edible, fished, harvested, cultivated.
4. Google Scholar was then searched for additional species to the list generated from the steps above using the following phrases, separated by semicolons:
    - exploited bivalve; harvest bivalve; commercial bivalve; artisanal bivalve; fishery bivalve; fisheries bivalve.
    - In each case, the first 20 pages of returned hits were examined.

Taxonomic Standardization.

Species were operationally assigned to genera using either the genus or subgenus designations, whichever represented the current lowest rank for the species. This approach increases sampling of hypothesized monophyletic groups that can be analyzed for their differences in traits (i.e. size, bathymetry, functional ecology). Eighty marine bivalve families were analyzed here, of the 100 currently recognized in MolluscaBase (as of Nov. 1, 2021). Following the time-calibrated, family-level phylogeny of Crouch et al.[1], phylogenetic positions of several families are uncertain (see summary in Supplementary text for Crouch et al.[1]). Therefore, Neilonellidae, Tindariidae, Sareptidae and Yoldiidae were folded into Malletiidae sensu lato; Phaseolidae, Bathyspinulidae, and Siliculidae were folded into Nuculanidae sensu lato; Nucinellidae was folded into Solemyidae; and Condylocardiidae was folded into Carditidae. The family Cardilidae (4 species) was omitted given its uncertain phylogenetic placement within Mactroidea [2]; the family Sportellidae (4 species) was omitted given its uncertain phylogenetic placement within Cyamioidea [3]. The Galeommatoidea (including Basterotiidae, Galeommatidae, Lasaeidae) are small-bodied and often commensal, and were thus omitted because of the corresponding sampling problems. The Teredinidae were omitted because shipping has severely altered their natural biogeography. Families with highly reduced shells or those that form part of a larger structure (tubes and crypts in Clavagellidae and Penicillidae) were also omitted, and we omitted. families known exclusively from the deep sea (e.g. Cetoconchidae, Protocuspidariidae).

Supplementary Table

Table S1. Exploitation is strongly associated with the total species richness in a taxonomic family, but not with family extinction history ($\hat{q}$), indicated by the coefficient estimates from Bayesian hierarchical regression models, after accounting for phylogenetic effect (quantified by the standard deviation of the response variable). Further details of all models and model comparisons are in Supplementary Code.

**Model Formula**	**Coefficient [mean estimate with 95% credible interval]**
exploitation (binary) ~ Family extinction history + phylogeny	Family extinction history = -3400 [-150000, 96000]
exploitation (binary) ~ Family Spp. Richness + phylogeny	Family Spp. Richness = 31 [1.8, 130]
No. exploited spp. ~ Family Spp. Richness + phylogeny	Family Spp. Richness = 0.55 [0.38, 0.72]
[Only for families with exploited species]<br>No. exploited spp. ~ Family Spp. Richness + phylogeny	Family Spp. Richness = 0.65 [0.48, 0.82]

Supplementary Figures

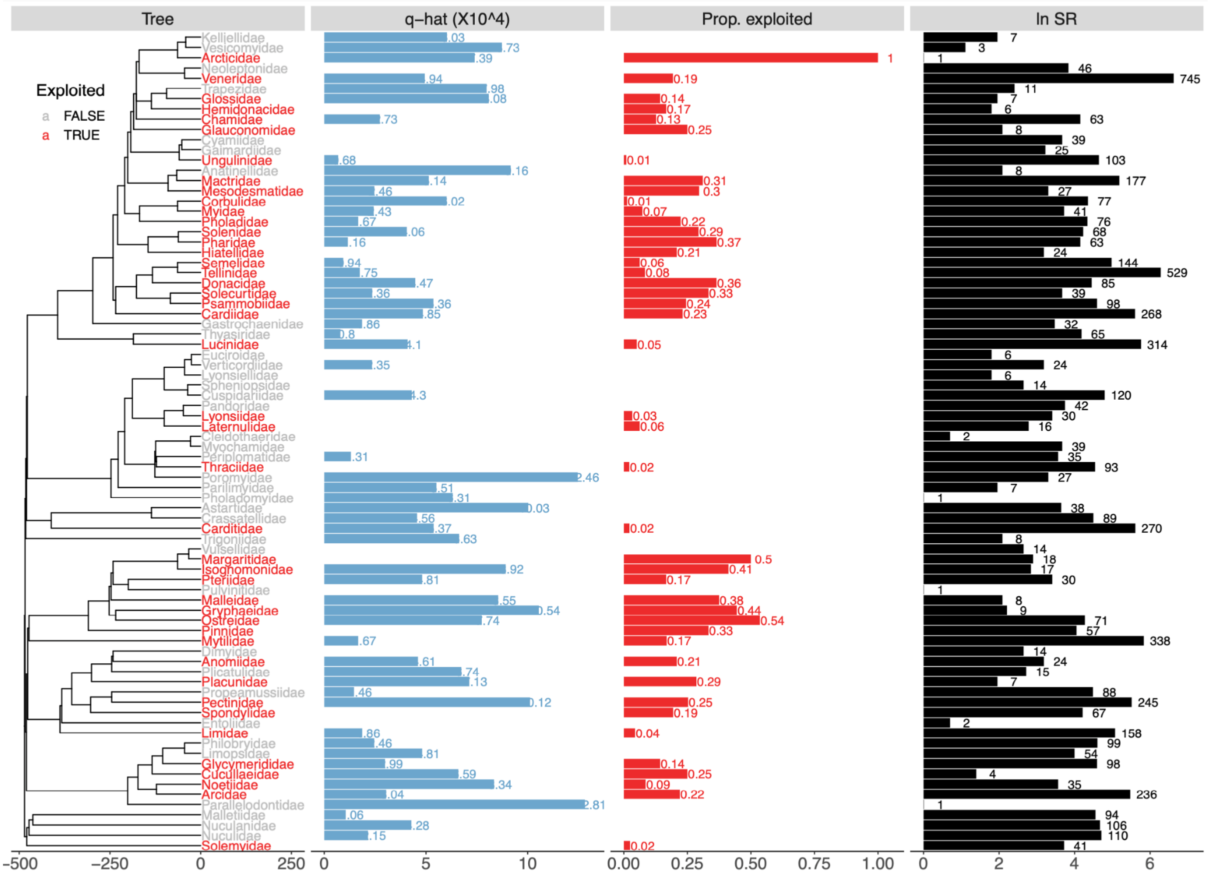

Figure S1. Family-level comparisons of extinction history (q-hat, representing the Cenozoic genus extinction rate), exploitation and species richness (with a natural logarithm transformation).

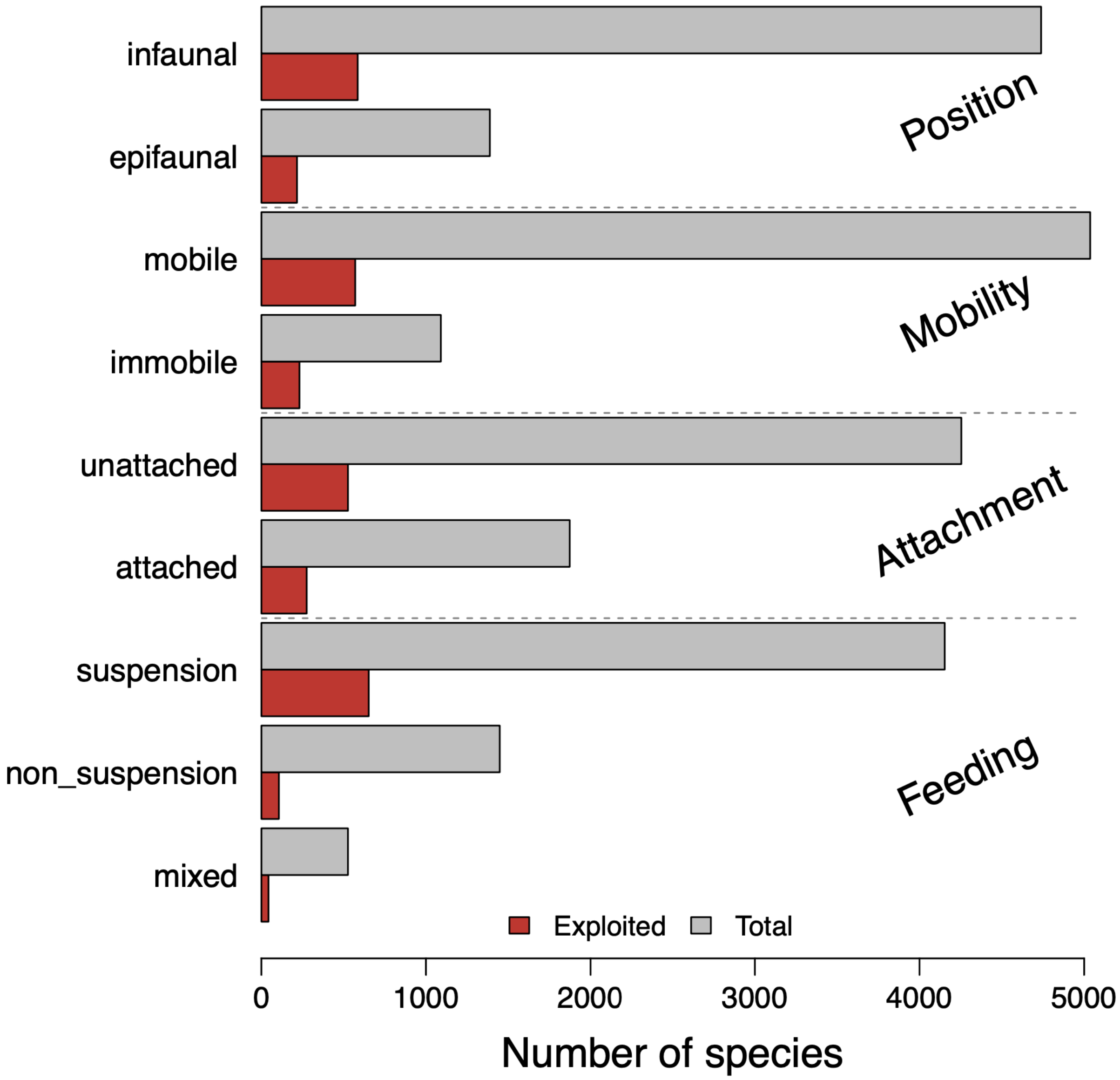

Figure S2. The number of exploited and all species in each functional category of shallow-marine bivalves.

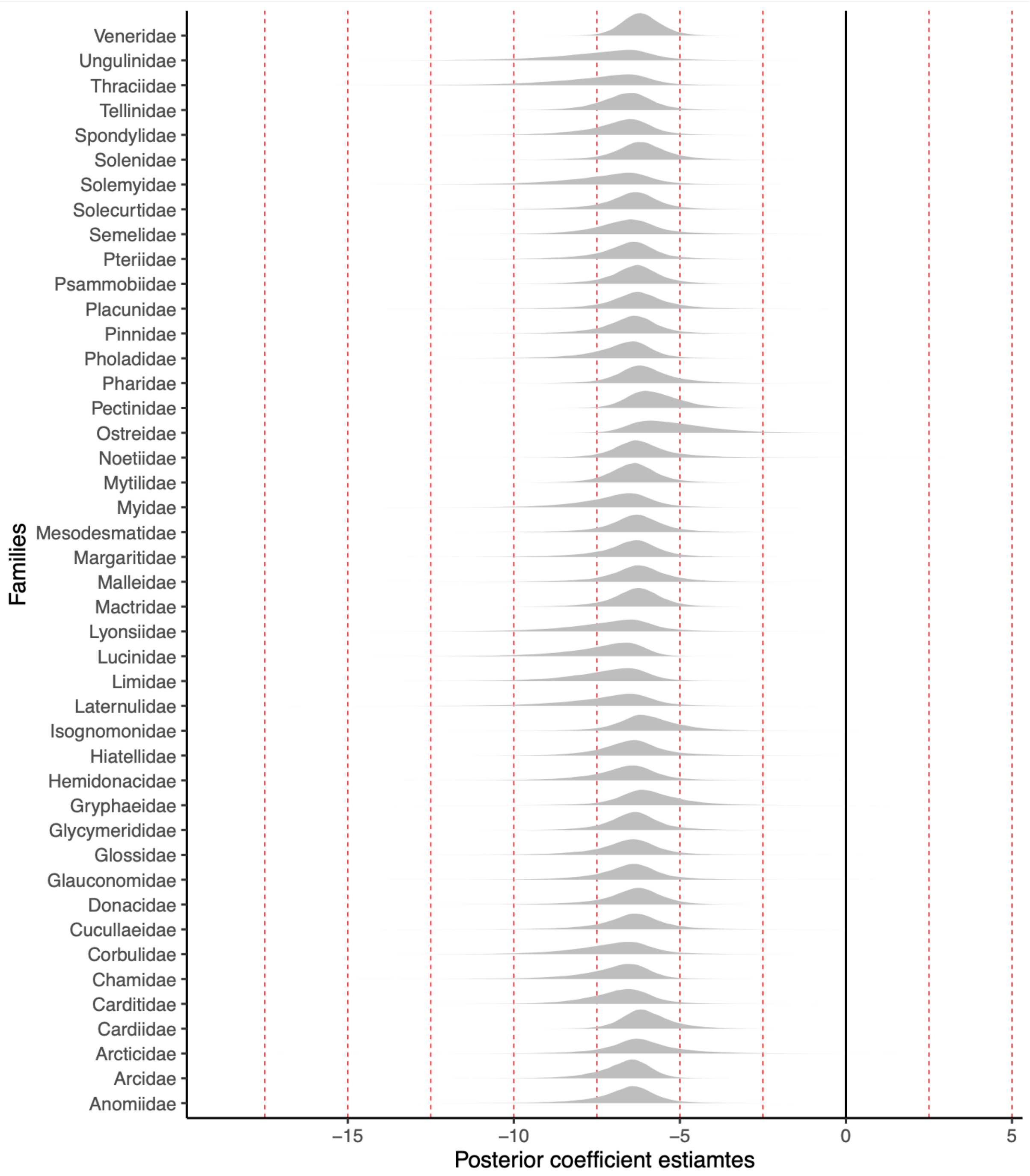

Figure S3. Exploited species consistently tend to have lower PERIL scores than unexploited species in the same family as shown by an additional model, where the posterior distributions were adjusted by family membership as a random effect on the coefficients: exploitation ~ PERIL + (PERIL|family). The mean estimated coefficient across all families was -6.88 [-8.48, -5.62] (sd = 1.09 [0.06, 2.62]), indicating that on average, species with lower PERIL scores have a higher probability of being exploited (consistent with Model 2, -6.32 [-7.08, -5.57], see also Figure 4a). See Supplementary Code for more details of the model.

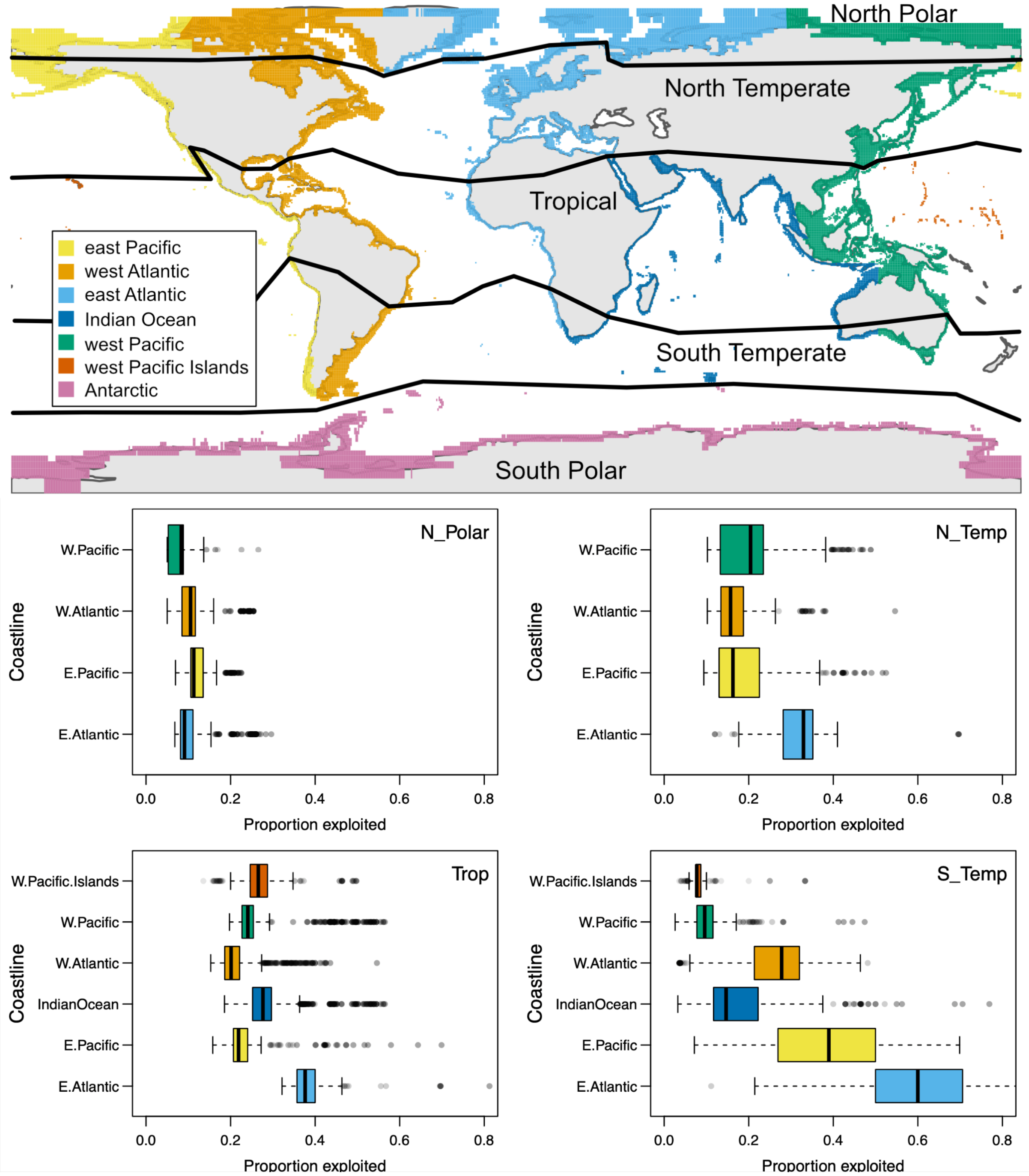

Figure S4. Top panel: Boundaries of climate and geographic zones used for classifying bivalve occurrence domains (i.e. climate-coastlines, modified and updated following protocol in Schumm et al.[4]). Bottom panel: The proportion of exploited species by 50x50 km grid cells varies among coastlines in the same climate zone (i.e. in the same panel; n = 6127 bivalves species, with 801 exploited). In particular, in both temperate zones, both eastern Pacific and eastern Atlantic (i.e. the western coasts of the continents) tend to have a higher proportion of their bivalve fauna being exploited species. The boxes represent the 25-75% quantiles (i.e. Q_1 and Q_3) of the data, the vertical black lines within the boxes represent the medians, and the horizontal dashed lines represent the the highest and lowest value excluding outliers which are defined as outside 1.5 times the interquartile range (IQR) above the upper quartile or below the lower quartile (Q_1 – 1.5 * IQR or Q_3 + 1.5 * IQR).

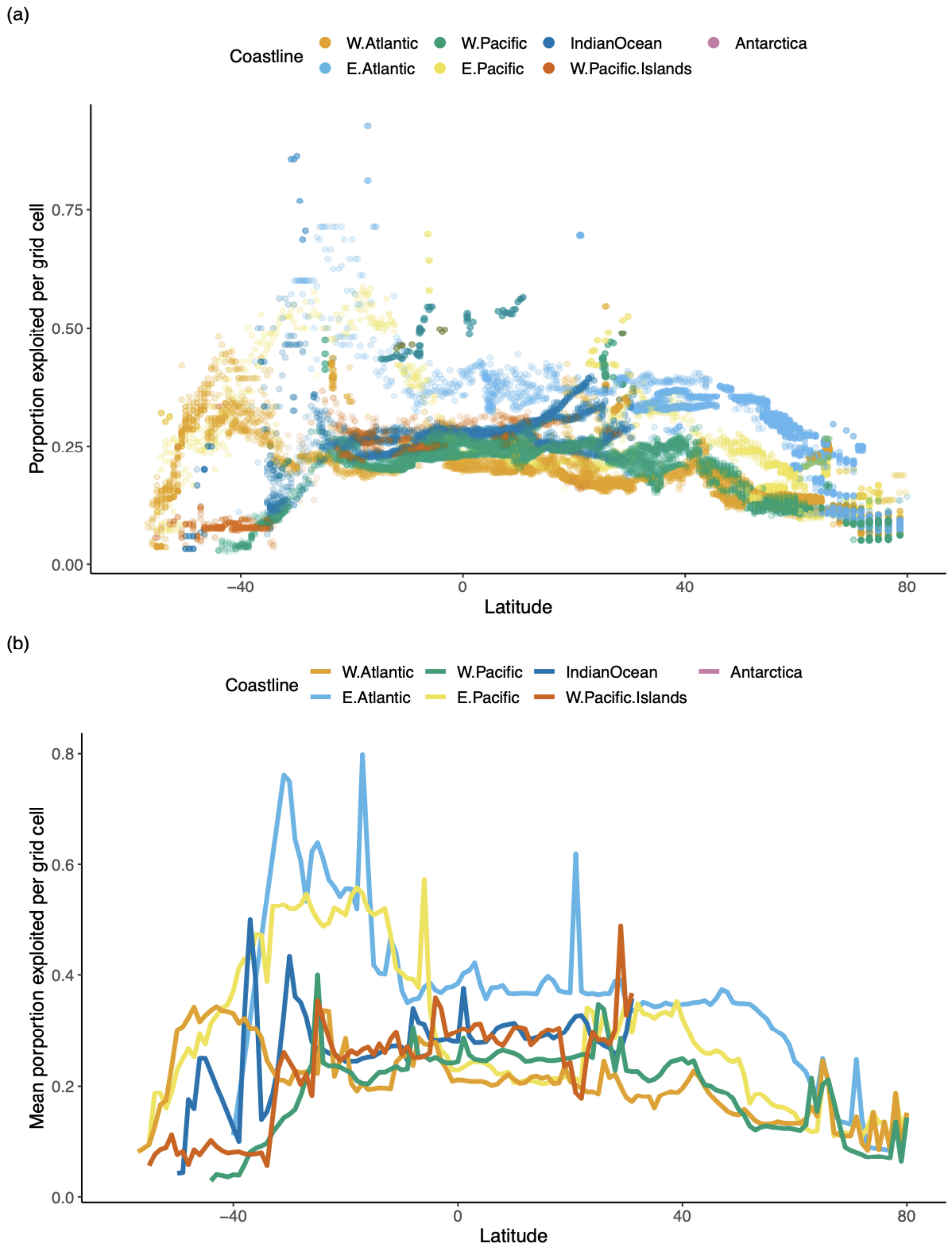

Figure S5. The proportion of exploited species by 50x50 km grid cells tends to be higher in temperate regions, especially along western coastlines. Data were illustrated for all grid cells in (a) and summarized as the mean by each degree of latitude in (b). The points and lines are

colored to indicate the major coastlines (similar to Fig. S4) and the color intensity in (a) reflects the density of points with similar data.

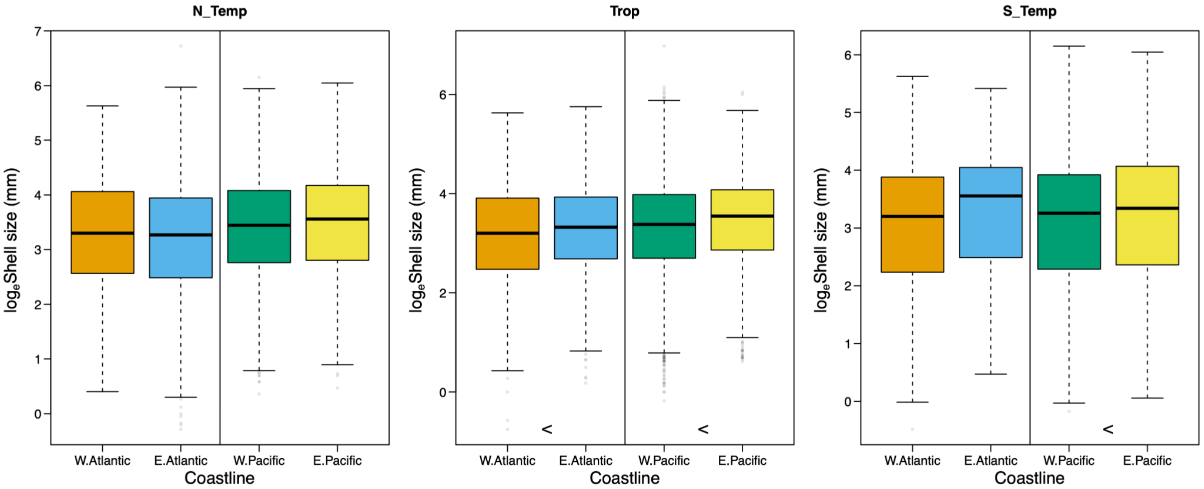

Figure S6. Although a higher proportion of exploited species are found on the eastern margins of ocean basins than on the western ones generally (Fig. 2d), eastern margins only have larger body sizes in the tropics (one-sided Wilcoxon rank-sum test: $p = 0.03$ for the Atlantic and $p = 0.001$ for the Pacific, $n = 6127$ bivalves species, with 801 exploited). In the southern temperate oceans, only the east Pacific fauna has larger-sized bivalve species than west Pacific ($p = 0.04$). Note that weaker contrasts were also found between west Atlantic and east Atlantic (<: $p = 0.075$) as well as east Pacific and west Pacific (<: p = 0.056). Here we use eastern boundaries of ocean basins to proxy higher-intensity upwelling vs. lower-intensity upwelling along western boundaries [89]. The boxes represent the 25-75% quantiles (i.e. Q_1 and Q_3) of the data, the horizontal black lines within the boxes represent the medians, and the vertical dashed lines represent the the highest and lowest value excluding outliers which are defined as outside 1.5 times the interquartile range (IQR) above the upper quartile or below the lower quartile ($Q_1 - 1.5 * IQR$ or $Q_3 + 1.5 * IQR$).

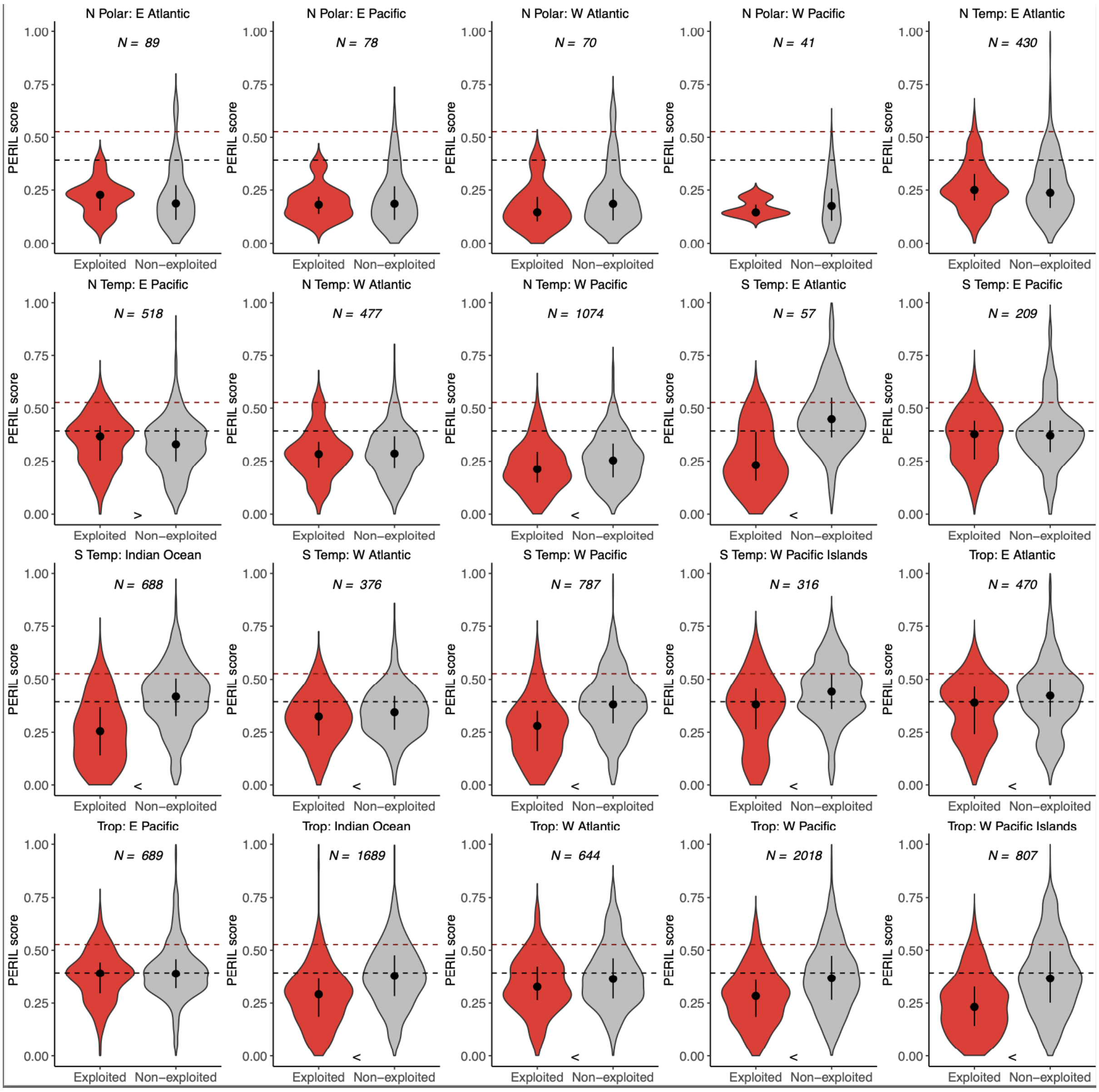

Figure S7. Not all regions have exploited species (red) with relatively low PERIL scores. Significant differences based on one-tail Wilcoxon rank-sum tests with $p<0.05$ are indicated by the symbols “>” or “<” depending on the direction of the difference. The global median and 80% quantile of PERIL in all bivalve species are indicated by the black and red dashed lines, respectively. A region is defined as a continental coastline within a broad climate zone (tropical, temperate or polar), with the number of occurring species indicated as '*N*='. Each violin reflects the density distribution of the species PERIL score with the median and 25-75% quantiles indicated by the black point and line, separated by whether the species has been reported as exploited in the literature (see data in Table S1).

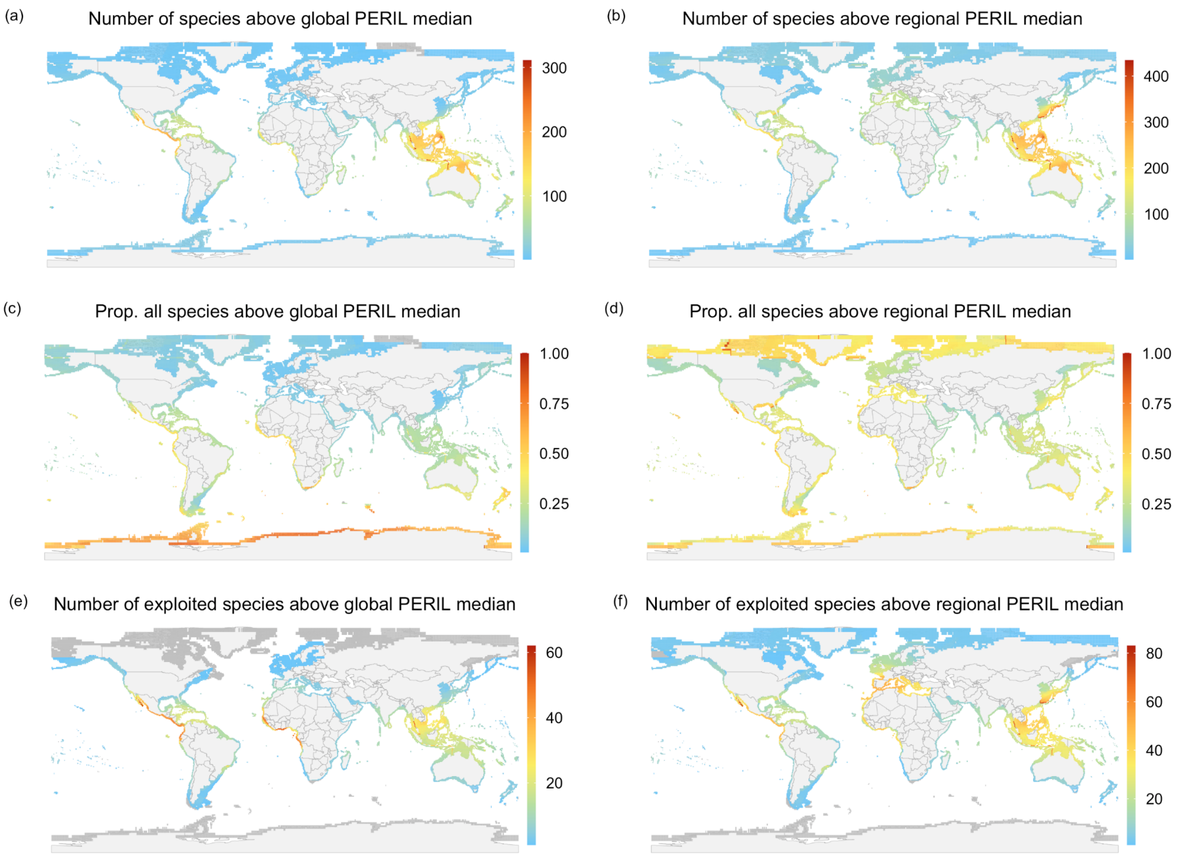

Figure S8. Richness peaks in the tropical Indo-Pacific bivalve diversity hotspot (see Figure 4) for both globally (a) and regionally (b) vulnerable bivalve species, defined as having PERIL scores above the global and regional medians respectively. In comparison, the proportion of globally vulnerable species (c) is relatively high in the Southern Hemisphere (Fig. S5, see also Collins et al.[5]). The richness of exploited vulnerable species (e-f) generally follows the patterns of all vulnerable species, but the number of regionally vulnerable species being exploited is also high in the Mediterranean Sea.

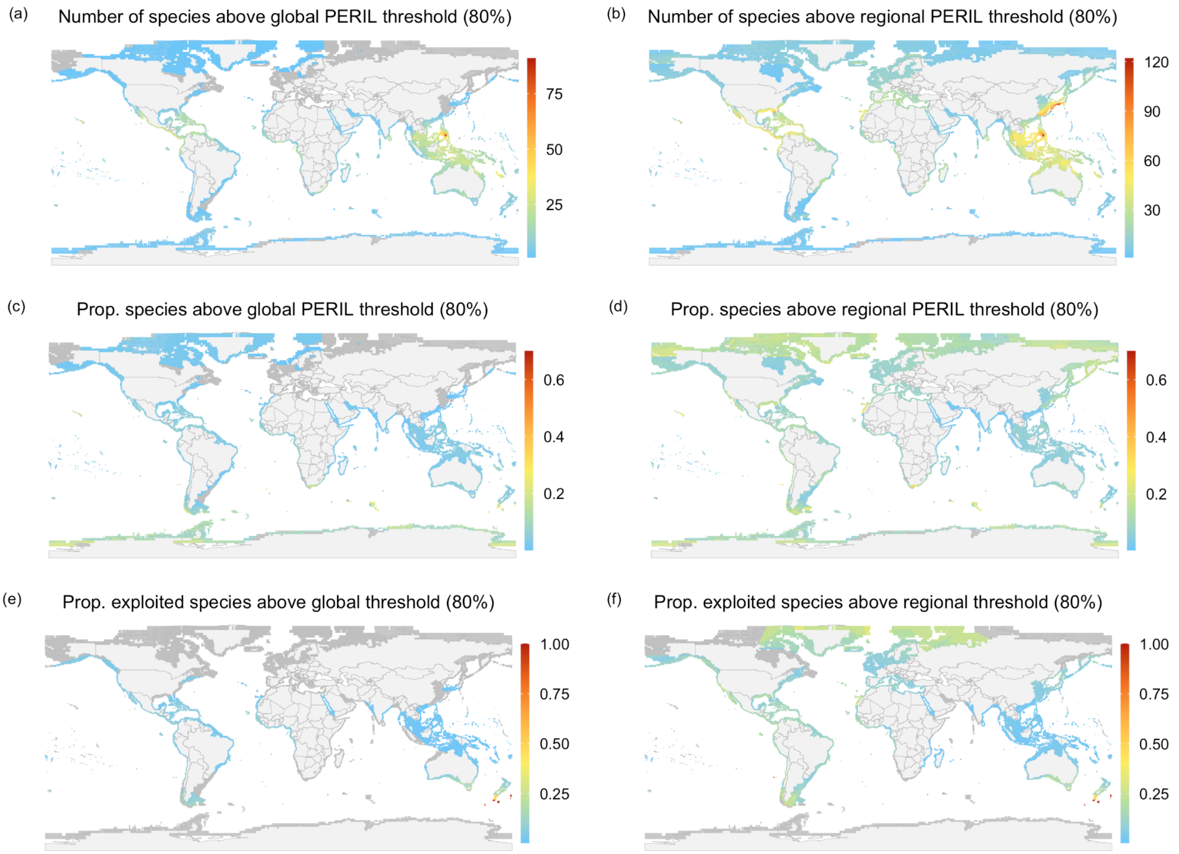

Figure S9. Based on the global 80% (a, c, e) and regional 80% (b, d, f) cutoffs, the number of all vulnerable bivalve species (a-b) peak in the tropical western Indo-Pacific bivalve diversity hotspot (see Fig. 2c), while the proportion of globally vulnerable species (c) is relatively high in the Southern Hemisphere (see also Collins et al. [5]). Higher proportions of exploited species vulnerable relative to the full bivalve fauna (f) are found in temperate regions than in the tropics.

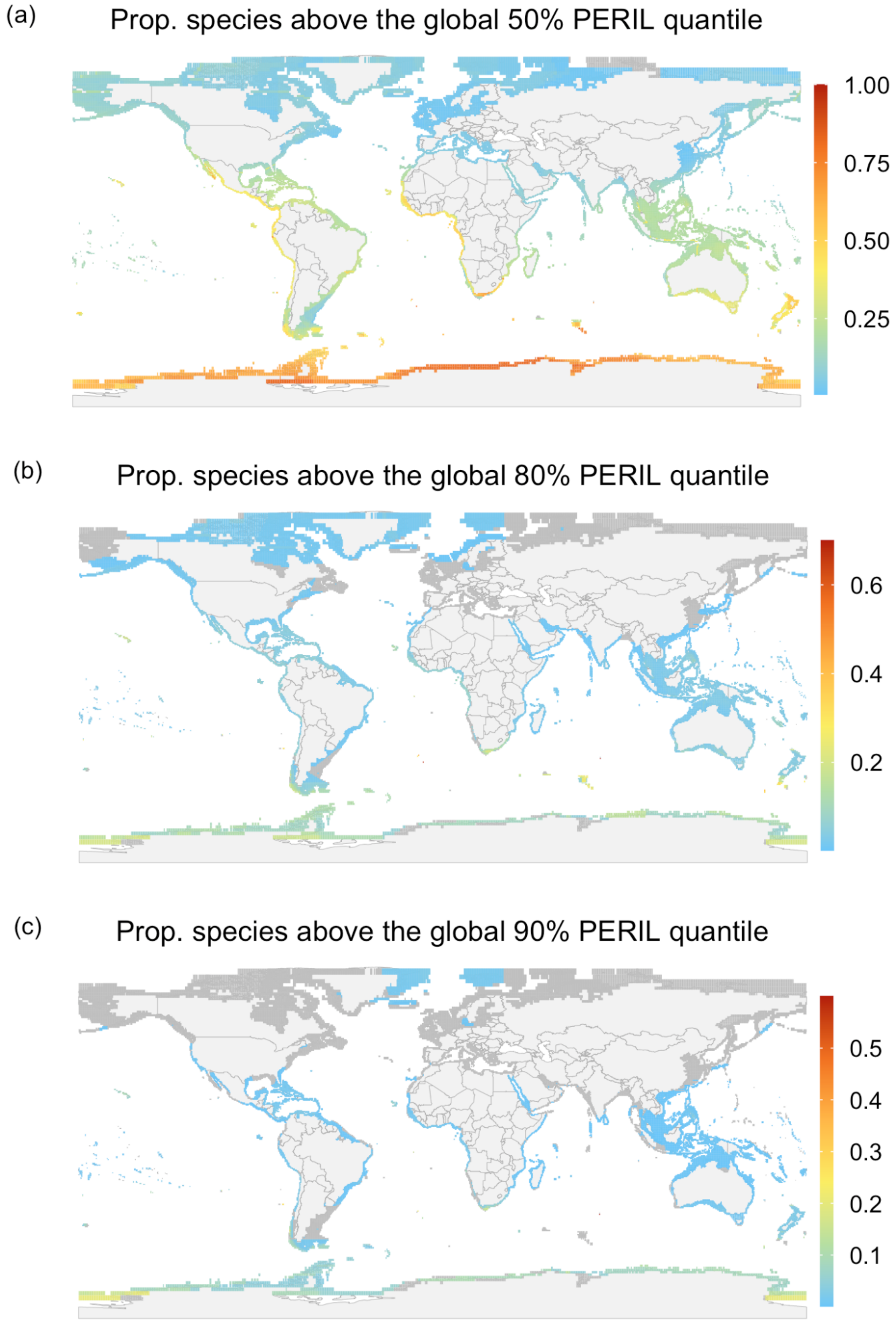

Figure S10. Comparisons of different global cutoffs (a: median, b: 80% quantile, c: 90% quantile) for defining vulnerable species.

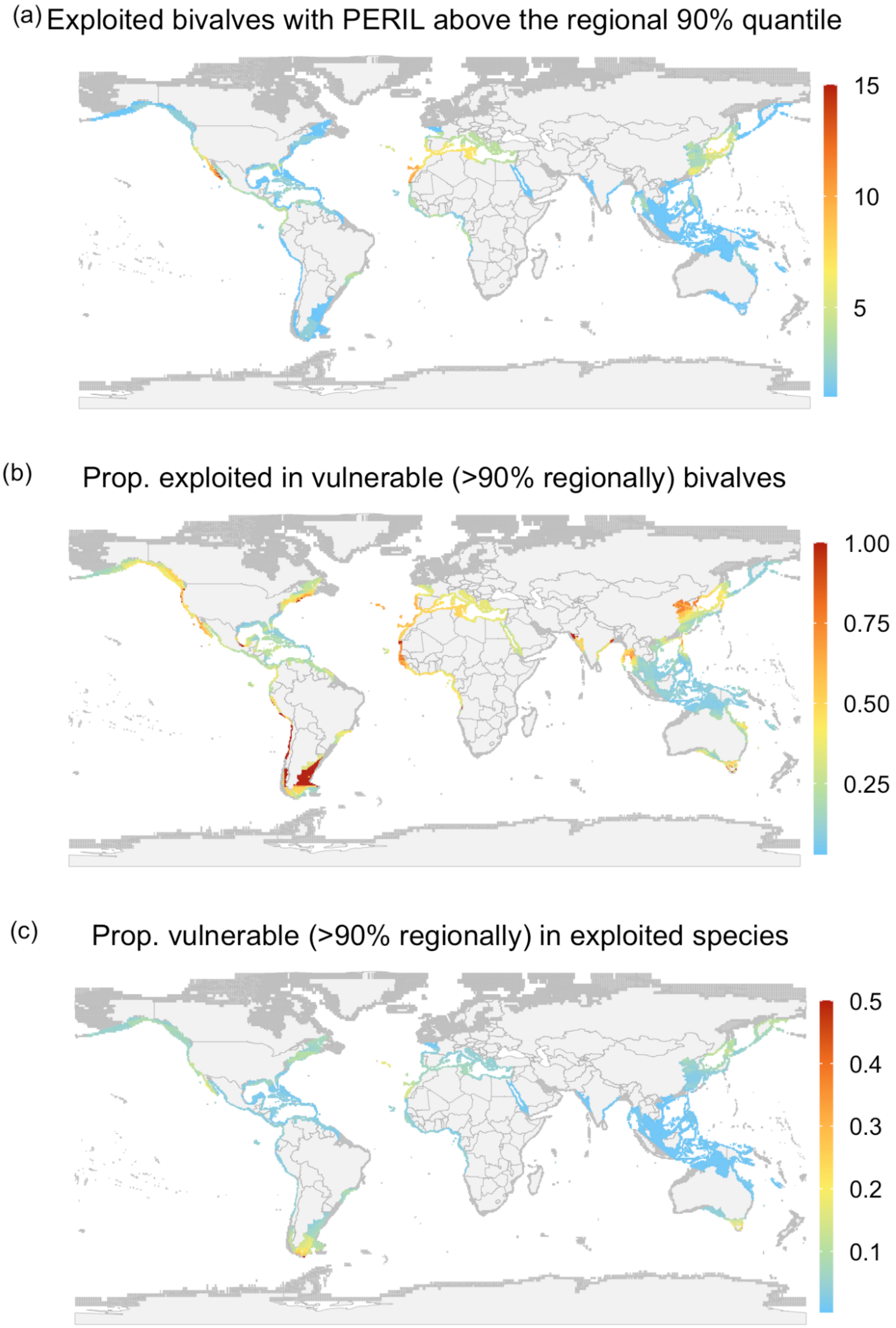

Figure S11. Distribution of vulnerable exploited species with PERIL scores above the regional 90% quantile.

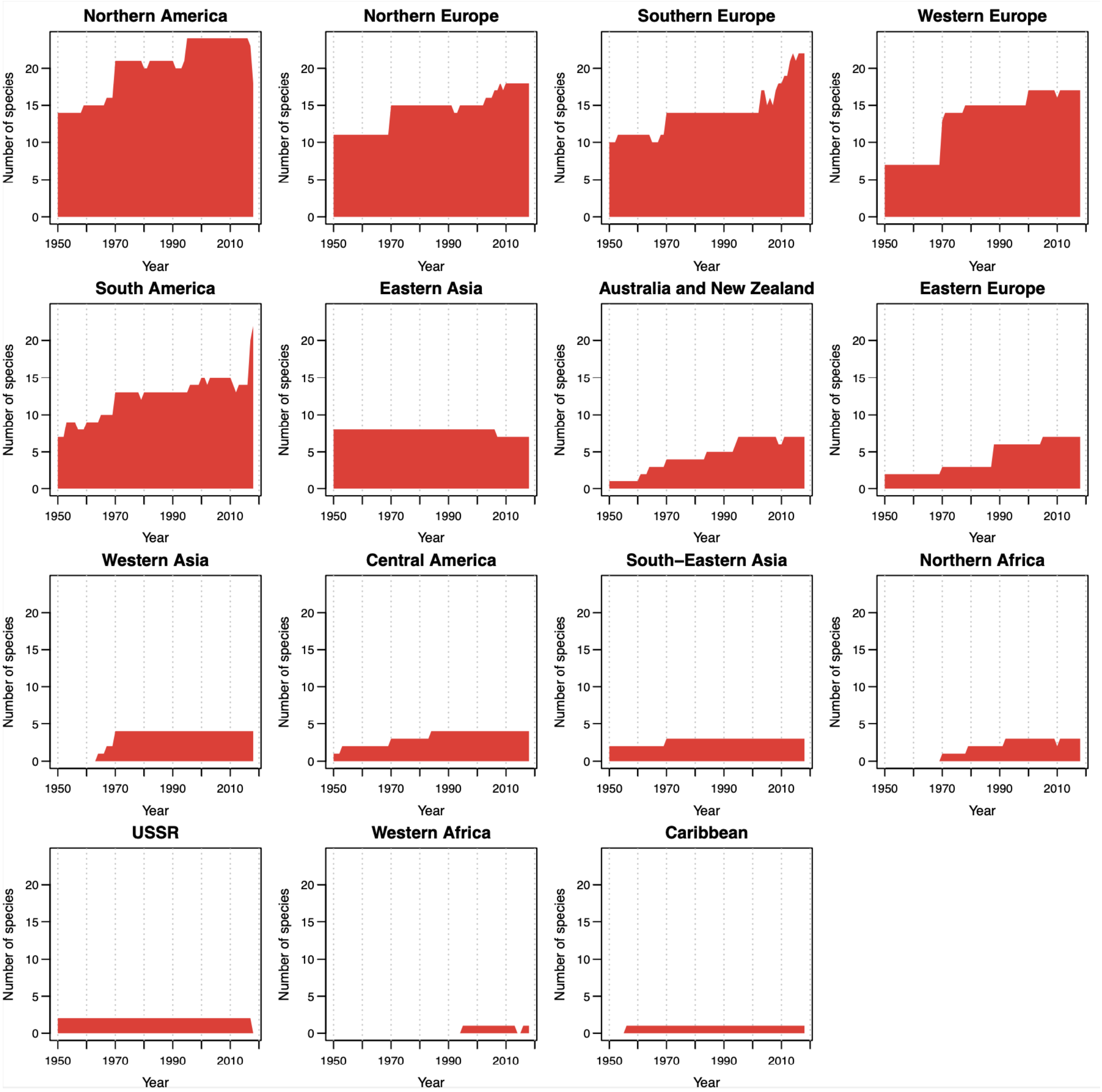

Figure S12. The number of species reported for each region during 1950-2018 in the FAO Capture Production Dataset (see Dataset S1).

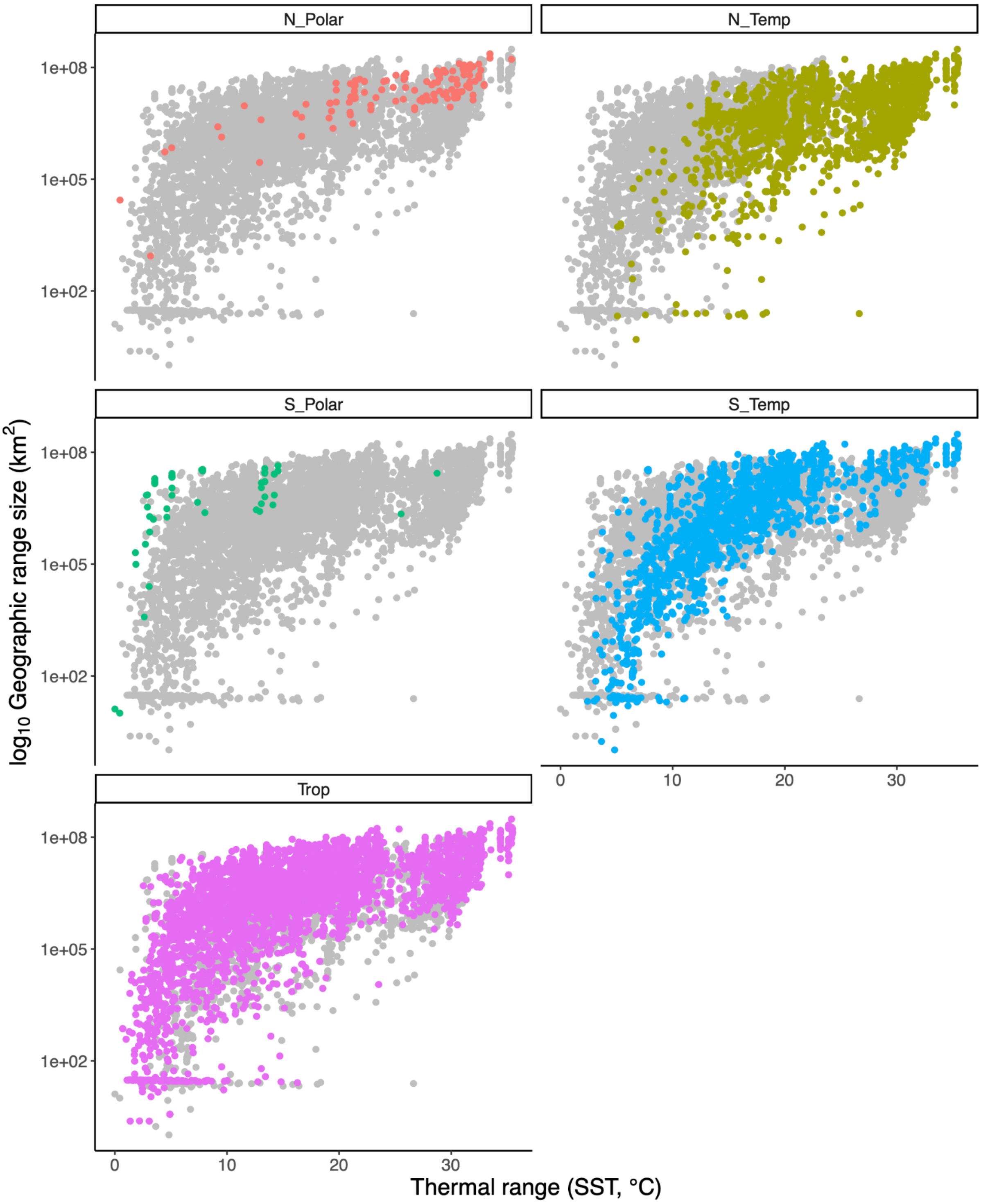

Figure S13. Widespread bivalve species (with large geographic ranges) are not necessarily eurythermal because many widespread species track a narrow range of sea-surface temperature across the large tropical or polar areas[6], as indicated by the colored points for the corresponding regions. The gray points at the background represent the global dataset.

Supplementary References

1. Crouch, N. M. A., Edie, S. M., Collins, K. S., Bieler, R. & Jablonski, D. Calibrating phylogenies assuming bifurcation or budding alters inferred macroevolutionary dynamics in a densely sampled phylogeny of bivalve families. *Proc. Royal Soc. B* **288**, 20212178 (2021).
2. Signorelli, J. H. & Raven, J. G. M. (Han). Current knowledge of the family Cardiliidae (Bivalvia, Mactroidea). *J. Paleontol.* **92**, 130–145 (2018).
3. Zelaya, D. G., Güller, M. & Ituarte, C. Filling a blank in bivalve taxonomy: an integrative analysis of Cyamioidea (Mollusca: Bivalvia). *Zool. J. Linn. Soc.* **190**, 558–591 (2019).
4. Schumm, M. *et al.* Common latitudinal gradients in functional richness and functional evenness across marine and terrestrial systems. *Proc. Royal Soc. B* **286**, 20190745 (2019).
5. Collins, K. S., Edie, S. M., Hunt, G., Roy, K. & Jablonski, D. Extinction risk in extant marine species integrating palaeontological and biodistributional data. *Proc. Royal Soc. B* **285**, 20181698 (2018).
6. Tomašových, A., Jablonski, D., Berke, S. K., Krug, A. Z. & Valentine, J. W. Nonlinear thermal gradients shape broad-scale patterns in geographic range size and can reverse Rapoport's rule. *Glob. Ecol. Biogeogr.* **24**, 157–167 (2015).
