# Supplementary material for: Diversity, distribution and intrinsic extinction vulnerability of exploited marine bivalves
Source: Nat Commun. 2023 Aug 15;14:4639. doi: 10.1038/s41467-023-40053-y (PMC10427664; doi:10.1038/s41467-023-40053-y)
Supplement: Supplementary file 2 — Description of Additional Supplementary Files [file 41467_2023_40053_MOESM2_ESM.pdf]

Description of Additional Supplementary Files

File name: **Supplementary Data 1**
Description: Excel workbook containing the following sheets:

- all_species_traits, contains all traits analyzed in the study for shallow-marine bivalve species.
- exploited_species, full list of exploited bivalve species discovered for these analyses
- exploited_references, full citations for exploited species.
- FAO_GlobalCaptureProduction, contains the annual production data from FAO, with taxonomic matches in the global bivalve trait dataset. (http://www.fao.org/fishery/statistics/en, accessed on August 6th, 2020)
- family_phylogeny, the NEWICK string for the time-calibrated phylogeny by Crouch et al.

File name: **Supplementary Data 2**
Description: A compressed txt file, that contains occurrences of bivalve species in 50x50 km grid cells. The columns are separated by commas, but the file cannot be fully read by Excel because the number of rows exceeds the limit of the program. Field Key:

- valid_species_id: unique integer ID linking species data across sheets.
- exploited: if TRUE, species is know to be exploited, see sheet: exploited_species.
- fao.prod: if TRUE, species is known from FAO Production Dataset, see sheet: FAO_GlobalCaptureProduction.
- peril: PERIL score of species.
- EAGid: unique integer ID of 50x50 km grid cell.
- climate: coarse climate of grid cell (see top panel of Fig. S4).
- coastline: coastline assignment of grid cell (see top panel of Fig. S4).
- ClimCoast: combination of climate and coastline of grid cell.

File name: **Supplementary Code**
Description: Analytical code including model description.
