# Supplementary material for: Diversity, distribution and intrinsic extinction vulnerability of exploited marine bivalves
Source: Nat Commun. 2023 Aug 15;14:4639. doi: 10.1038/s41467-023-40053-y (PMC10427664; doi:10.1038/s41467-023-40053-y)
Supplement: Supplementary file 5 — Supplementary Code [file 41467_2023_40053_MOESM5_ESM.pdf]

# Supplementary Code

Diversity, distribution and intrinsic extinction vulnerability of exploited marine bivalves

## Contents

**Packages** **2**

**Data** **2**
Traits . . . . . 2
Phylogeny . . . . . 2
Occurrences . . . . . 2

**Phylogenetic distribution of exploited species** **2**
Phylogenetic signal . . . . . 2
Total species richness . . . . . 3

**Biological traits of exploited species** **10**
Model comparison - LOO . . . . . 12
Model comparison - LOGO . . . . . 13
Model 1: traits . . . . . 13
Model 2: traits+family . . . . . 14
Model 3: traits+phylogeny . . . . . 15
Trait distributions . . . . . 16

**Intrinsic vulnerability of exploited species** **17**
Family extinction history . . . . . 17
PERIL score . . . . . 19

**Biogeography of exploited species** **25**

# Packages

```
library(tidyverse) # for data processing
library(brms) # for Bayesian models
library(sf) # for spatial analyses
library(readxl) # for reading .xlsx files
```

# Data

## Traits

```
# bivalve species data
trait<-readxl::read_xlsx(paste(data_folder, "Supplementary Data 1.xlsx", sep=""),
    sheet="all_species_traits", na=c('NA','')) %>%
  mutate_all(type.convert,as.is=TRUE)

# make the family dataset
fam.eis<-trait %>%
    group_by(family) %>%
    summarise(sr=length(family),
              exploited=sum(1*exploited),
              ex_prop=exploited/sr,
              qhatH=max(qhat, na.rm=TRUE)) %>%
    mutate(exploited_bin=(exploited>0)) %>%
    as.data.frame()
```

## Phylogeny

```
tree_string <- readxl::read_xlsx(paste(data_folder, 'Supplementary Data 1.xlsx', sep=""),
  sheet='family_phylogeny', col_names=FALSE)
fam.tree  <- ape::read.tree(text=unlist(tree_string))
fam.tree<-ape::drop.tip(fam.tree, which(!fam.tree$tip.label %in% trait$family))
```

## Occurrences

```
###########################################################################
# species occurrence
spp.occ<-read.csv(paste(data_folder, "Supplementary Data 2.txt", sep=""))
```

# Phylogenetic distribution of exploited species

## Phylogenetic signal

Phylogenetic signal was identified in two ways: a) the presence or absence of any exploited species in a family using phylo-D (for a binary trait), and b) the proportion of exploited species in a family (i.e. a continuous variable) using Pagel's (for a continuous trait).

```
## phylo signal of having exploited species or not (binary variable)
fam.eis.bin<-fam.eis %>%
    mutate(exploited=1*(exploited>0)) %>%
    select(family, exploited) %>%
    unique()
```

```r
# phylo signal using phylo-d
caper::phylo.d(data=as.data.frame(fam.eis.bin), phy=fam.tree,
               names.col=family, binvar=exploited,
               permut = 1000, rnd.bias=NULL)


## phylo signal of proportion of exploited species
x<-fam.eis$ex_prop
names(x)<-fam.eis$family

# all families
set.seed(2022)
phytools::phylosig(tree=fam.tree, x=x[fam.tree$tip.label], method="lambda", test=TRUE)
```

## Total species richness

The number of exploited species in a family is expected to correlate with the total number of species; the former cannot exceed the latter, and the total species richness varies among families.

Both the total number of species in a family and its number of exploited species are skewed, so each was transformed prior to modeling using the natural logarithm. Those families lacking exploited species were transformed as `log(exploited+1)`.

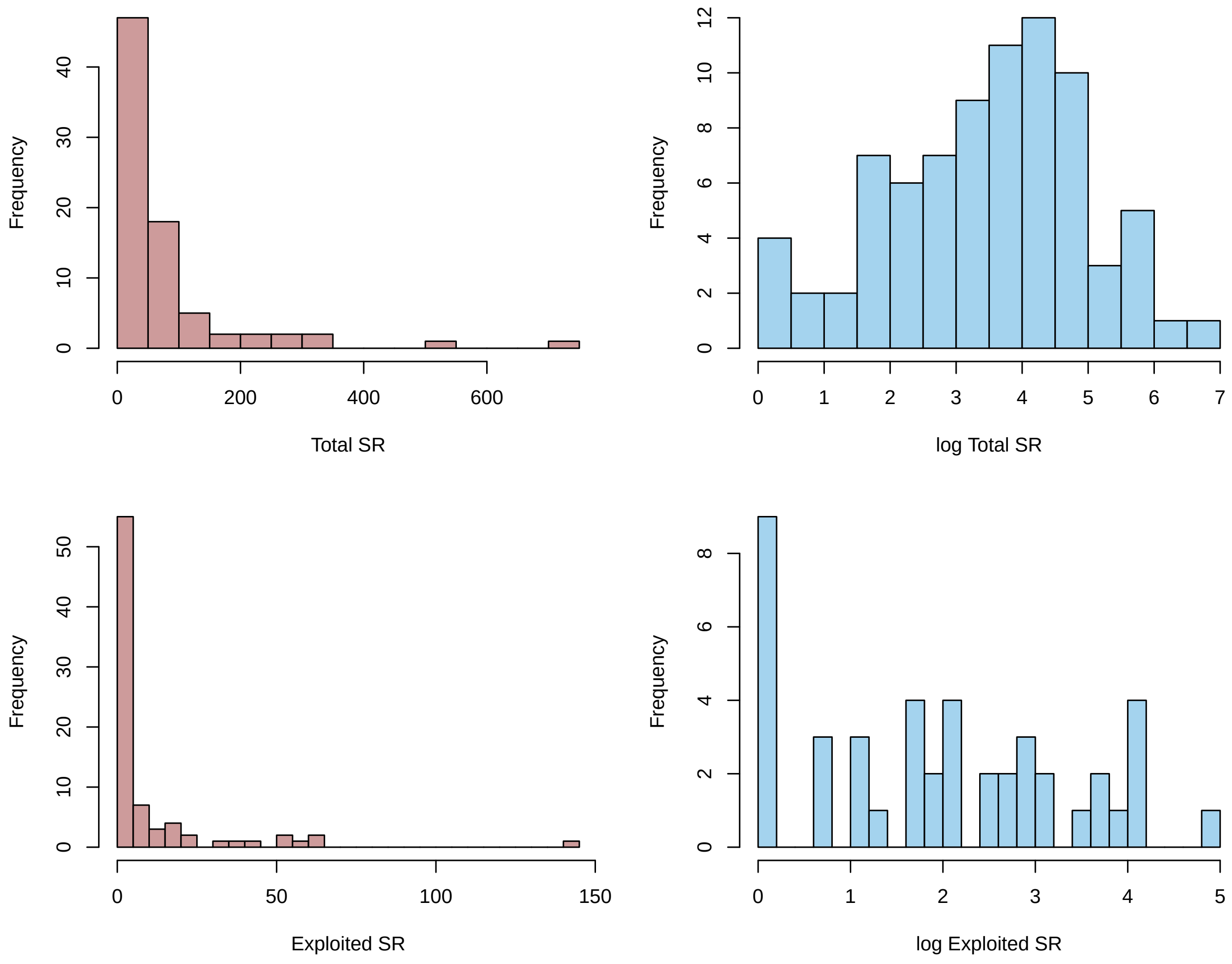

We modeled the relationship between species richness in a family and exploitation in three ways:

- as the number of exploited species in a family vs. its total number of species (`fam_sr_brm_1.[X]` below);
- as above, but only for families containing exploited species (`fam_sr_brm_2.[X]`);
- as the presence or absensce of exploited species in a family vs. its total number of species in the family (`fam_sr_brm_3.[X]`);

where each relationship was modeled: - across the dataset (i.e. without a family-level offset, or random effect; `[model].1` below); - with a family-level offset accounting for potential phylogenetic structure as the variance-covariance matrix from the family-level phylogeny (`[model].2`).

### Across all families

```
# individual-level model
fam_sr_brm_1.1<-brm(log(exploited+1) ~ log(sr),
    data=fam.eis,
    family = gaussian(),
    iter=iter, chains=chains, cores=cores, warmup=warmup, thin=thin, seed=seed)

# group-level model
fam_sr_brm_1.2<-brm(log(exploited+1) ~ log(sr) + (1|gr(family, cov=A)),
    data=fam.eis,
    data2=list(A=A),
    family = gaussian(),
    iter=iter, chains=chains, cores=cores, warmup=warmup, thin=thin, seed=seed)
```

The phylogenetically informed model performs better than the individual-level model ($\Delta$ELPD $> 2$):

```
mod_comp<-readRDS(paste(result_folder,"SR_EIS_model_loo.rds", sep=""))
mod_comp[[1]]
```

```
## Output of model 'fam_sr_brm_1.1':
##
## Computed from 50000 by 80 log-likelihood matrix
##
##          Estimate  SE
## elpd_loo   -127.6 4.0
## p_loo         2.3 0.3
## looic       255.2 8.0
## ------
## Monte Carlo SE of elpd_loo is 0.0.
##
## All Pareto k estimates are good (k < 0.5).
## See help('pareto-k-diagnostic') for details.
##
## Output of model 'fam_sr_brm_1.2':
##
## Computed from 50000 by 80 log-likelihood matrix
##
##          Estimate   SE
## elpd_loo   -120.3  5.1
## p_loo        29.0  2.8
## looic       240.6 10.1
## ------
## Monte Carlo SE of elpd_loo is NA.
```

```
##
## Pareto k diagnostic values:
##                          Count Pct.    Min. n_eff
## (-Inf, 0.5]   (good)     28    35.0%   2215
##  (0.5, 0.7]   (ok)       44    55.0%   576
##    (0.7, 1]   (bad)       8    10.0%   159
##    (1, Inf)   (very bad)  0     0.0%   <NA>
## See help('pareto-k-diagnostic') for details.
##
## Model comparisons:
##                elpd_diff se_diff
## fam_sr_brm_1.2  0.0       0.0
## fam_sr_brm_1.1 -7.3       3.6
```

**Individual-level model** The number of exploited species is positively correlated with total species richness among families.

```
mod<-readRDS(paste(result_folder,"fam_sr_EIS.rds", sep=""))
mod
```

```
##  Family: gaussian
##   Links: mu = identity; sigma = identity
## Formula: log(exploited + 1) ~ log(sr)
##    Data: fam.eis (Number of observations: 80)
##   Draws: 5 chains, each with iter = 15000; warmup = 5000; thin = 1;
##          total post-warmup draws = 50000
##
## Population-Level Effects:
##           Estimate Est.Error l-95% CI u-95% CI Rhat Bulk_ESS Tail_ESS
## Intercept    -0.68      0.33    -1.34    -0.03 1.00    49163    37682
## logsr         0.56      0.09     0.38     0.73 1.00    48827    36872
##
## Family Specific Parameters:
##       Estimate Est.Error l-95% CI u-95% CI Rhat Bulk_ESS Tail_ESS
## sigma     1.18      0.10     1.01     1.39 1.00    49153    38302
##
## Draws were sampled using sampling(NUTS). For each parameter, Bulk_ESS
## and Tail_ESS are effective sample size measures, and Rhat is the potential
## scale reduction factor on split chains (at convergence, Rhat = 1).
```

**Phylogenetically informed model** The number of exploited species is positively correlated with total species richness among families, with phylogenetic covariance accounting for a small proportion of the total residual variance in the relationship (compare individual-level `sigma` to Group-level `sd(Intercept)` below).

```
mod<-readRDS(paste(result_folder,"fam_sr_EIS_phylo.rds", sep=""))
mod
```

```
##  Family: gaussian
##   Links: mu = identity; sigma = identity
## Formula: log(exploited + 1) ~ log(sr) + (1 | gr(family, cov = A))
##    Data: fam.eis (Number of observations: 80)
##   Draws: 5 chains, each with iter = 15000; warmup = 5000; thin = 1;
##          total post-warmup draws = 50000
##
## Group-Level Effects:
```

```
## ~family (Number of levels: 80)
##               Estimate Est.Error l-95% CI u-95% CI Rhat Bulk_ESS Tail_ESS
## sd(Intercept)     0.04      0.01     0.02     0.06 1.00     6783     7966
##
## Population-Level Effects:
##           Estimate Est.Error l-95% CI u-95% CI Rhat Bulk_ESS Tail_ESS
## Intercept    -0.87      0.41    -1.68    -0.08 1.00    28151    28166
## logsr         0.55      0.09     0.38     0.72 1.00    41672    35186
##
## Family Specific Parameters:
##       Estimate Est.Error l-95% CI u-95% CI Rhat Bulk_ESS Tail_ESS
## sigma     0.87      0.13     0.62     1.13 1.00     6838     6864
##
## Draws were sampled using sampling(NUTS). For each parameter, Bulk_ESS
## and Tail_ESS are effective sample size measures, and Rhat is the potential
## scale reduction factor on split chains (at convergence, Rhat = 1).
```

### Across only families with exploited species

```
# individual-level model
fam_sr_brm_2.1<-brm(log(exploited+1) ~ log(sr),
    data=filter(fam.eis, exploited>0),
    family = gaussian(),
    iter=iter, chains=chains, cores=cores, warmup=warmup, thin=thin, seed=seed)

# group-level model
fam_sr_brm_2.2<-brm(log(exploited+1) ~ log(sr) + (1|gr(family, cov=A)),
    data=filter(fam.eis, exploited>0),
    data2=list(A=A),
    family = gaussian(),
    iter=iter, chains=chains, cores=cores, warmup=warmup, thin=thin, seed=seed)
```

The phylogenetically informed model performs better than the individual-level model ($\Delta$ELPD $> 2$), but with lower convergence rate (based on the Pareto k diagnostic values).

```
mod_comp[[2]]
```

```
## Output of model 'fam_sr_brm_2.1':
##
## Computed from 50000 by 44 log-likelihood matrix
##
##          Estimate  SE
## elpd_loo    -56.5 4.4
## p_loo         2.6 0.6
## looic       113.0 8.7
## ------
## Monte Carlo SE of elpd_loo is 0.0.
##
## All Pareto k estimates are good (k < 0.5).
## See help('pareto-k-diagnostic') for details.
##
## Output of model 'fam_sr_brm_2.2':
##
## Computed from 50000 by 44 log-likelihood matrix
##
```

```
##          Estimate  SE
## elpd_loo    -46.0 4.7
## p_loo        30.5 3.8
## looic        91.9 9.4
## ------
## Monte Carlo SE of elpd_loo is NA.
##
## Pareto k diagnostic values:
##                          Count Pct.    Min. n_eff
## (-Inf, 0.5]   (good)      4     9.1%   64
##  (0.5, 0.7]   (ok)       13    29.5%   6
##    (0.7, 1]   (bad)      27    61.4%   0
##    (1, Inf)   (very bad)  0     0.0%   <NA>
## See help('pareto-k-diagnostic') for details.
##
## Model comparisons:
##                 elpd_diff se_diff
## fam_sr_brm_2.2   0.0       0.0
## fam_sr_brm_2.1 -10.5       2.1
```

**Individual-level model** The number of exploited species is correlated with total species richness among families with at least one exploited species.

```
mod<-readRDS(paste(result_folder,"fam_sr_EIS_EISfamily.rds", sep=""))
mod
```

```
##  Family: gaussian
##   Links: mu = identity; sigma = identity
## Formula: log(exploited + 1) ~ log(sr)
##    Data: filter(fam.eis, exploited > 0) (Number of observations: 44)
##   Draws: 5 chains, each with iter = 15000; warmup = 5000; thin = 1;
##          total post-warmup draws = 50000
##
## Population-Level Effects:
##           Estimate Est.Error l-95% CI u-95% CI Rhat Bulk_ESS Tail_ESS
## Intercept    -0.21      0.38    -0.97     0.54 1.00    45001    34961
## logsr         0.63      0.09     0.44     0.81 1.00    44249    35067
##
## Family Specific Parameters:
##       Estimate Est.Error l-95% CI u-95% CI Rhat Bulk_ESS Tail_ESS
## sigma     0.86      0.10     0.70     1.08 1.00    40924    34099
##
## Draws were sampled using sampling(NUTS). For each parameter, Bulk_ESS
## and Tail_ESS are effective sample size measures, and Rhat is the potential
## scale reduction factor on split chains (at convergence, Rhat = 1).
```

**Phylogenetically informed model** The number of exploited species is correlated with total species richness among families, with phylogenetic covariance accounting for a small proportion of the total residual variance in the relationship.

```
mod<-readRDS(paste(result_folder,"fam_sr_EIS_EISfamily_phylo.rds", sep=""))
mod
```

```
##  Family: gaussian
##   Links: mu = identity; sigma = identity
```

```
## Formula: log(exploited + 1) ~ log(sr) + (1 | gr(family, cov = A))
##    Data: filter(fam.eis, exploited > 0) (Number of observations: 44)
##   Draws: 5 chains, each with iter = 15000; warmup = 5000; thin = 1;
##          total post-warmup draws = 50000
##
## Group-Level Effects:
## ~family (Number of levels: 44)
##               Estimate Est.Error l-95% CI u-95% CI Rhat Bulk_ESS Tail_ESS
## sd(Intercept)     0.04      0.01     0.01     0.06 1.01     1334      890
##
## Population-Level Effects:
##           Estimate Est.Error l-95% CI u-95% CI Rhat Bulk_ESS Tail_ESS
## Intercept    -0.49      0.45    -1.40     0.35 1.01     1066     5591
## logsr         0.65      0.09     0.48     0.82 1.00     7267     9493
##
## Family Specific Parameters:
##       Estimate Est.Error l-95% CI u-95% CI Rhat Bulk_ESS Tail_ESS
## sigma     0.49      0.20     0.16     0.88 1.02      272      102
##
## Draws were sampled using sampling(NUTS). For each parameter, Bulk_ESS
## and Tail_ESS are effective sample size measures, and Rhat is the potential
## scale reduction factor on split chains (at convergence, Rhat = 1).
```

### Presence of exploitation in a family

```
# individual-level model
fam_sr_brm_3.1<-brm(exploited_bin ~ log(sr),
    data=fam.eis,
    family = bernoulli(link="logit"),
    iter=iter, chains=chains, cores=cores, warmup=warmup, thin=thin, seed=seed)

# group-level model
fam_sr_brm_3.2<-brm(exploited_bin ~ log(sr) + (1|gr(family, cov=A)),
    data=fam.eis, data2=list(A=A),
    family = bernoulli(link="logit"),
    iter=iter, chains=chains, cores=cores, warmup=warmup, thin=thin, seed=seed)
```

The phylogenetically informed model performs better than the individual-level model ($\Delta$ELPD $> 2$), but with lower convergence rate (based on the Pareto k diagnostic values).

```
mod_comp[[3]]
```

```
## Output of model 'fam_sr_brm_3.1':
##
## Computed from 50000 by 80 log-likelihood matrix
##
##          Estimate  SE
## elpd_loo    -52.1 3.2
## p_loo         2.0 0.3
## looic       104.3 6.5
## ------
## Monte Carlo SE of elpd_loo is 0.0.
##
## All Pareto k estimates are good (k < 0.5).
## See help('pareto-k-diagnostic') for details.
```

```
##
## Output of model 'fam_sr_brm_3.2':
##
## Computed from 50000 by 80 log-likelihood matrix
##
##          Estimate  SE
## elpd_loo    -11.4 1.4
## p_loo         9.7 1.2
## looic        22.8 2.7
## ------
## Monte Carlo SE of elpd_loo is NA.
##
## Pareto k diagnostic values:
##                          Count Pct.    Min. n_eff
## (-Inf, 0.5]   (good)      0     0.0%   <NA>
##  (0.5, 0.7]   (ok)        0     0.0%   <NA>
##    (0.7, 1]   (bad)      43    53.8%   17
##    (1, Inf)   (very bad) 37    46.2%   9
## See help('pareto-k-diagnostic') for details.
##
## Model comparisons:
##                elpd_diff se_diff
## fam_sr_brm_3.2   0.0       0.0
## fam_sr_brm_3.1 -40.7       2.9
```

**Individual-level model** Exploited families tend to have higher total species richness.

```
mod<-readRDS(paste(result_folder,"fam_sr_EISbin.rds", sep=""))
mod
```

```
##  Family: bernoulli
##   Links: mu = logit
## Formula: exploited_bin ~ log(sr)
##    Data: fam.eis (Number of observations: 80)
##   Draws: 5 chains, each with iter = 15000; warmup = 5000; thin = 1;
##          total post-warmup draws = 50000
##
## Population-Level Effects:
##           Estimate Est.Error l-95% CI u-95% CI Rhat Bulk_ESS Tail_ESS
## Intercept    -1.63      0.67    -3.01    -0.38 1.00    40022    30910
## logsr         0.54      0.18     0.20     0.92 1.00    38879    31655
##
## Draws were sampled using sampling(NUTS). For each parameter, Bulk_ESS
## and Tail_ESS are effective sample size measures, and Rhat is the potential
## scale reduction factor on split chains (at convergence, Rhat = 1).
```

**Phylogenetically informed model** Exploited families tend to have higher total species richness, with wide range in uncertainty for the effect of phylogenetic covariance on family-level differences.

```
mod<-readRDS(paste(result_folder,"fam_sr_EISbin_phylo.rds", sep=""))
print(mod, digits=3)
```

```
##  Family: bernoulli
##   Links: mu = logit
## Formula: exploited_bin ~ log(sr) + (1 | gr(family, cov = A))
```

```
##    Data: fam.eis (Number of observations: 80)
##   Draws: 5 chains, each with iter = 15000; warmup = 5000; thin = 1;
##          total post-warmup draws = 50000
##
## Group-Level Effects:
## ~family (Number of levels: 80)
##               Estimate Est.Error l-95% CI u-95% CI  Rhat Bulk_ESS Tail_ESS
## sd(Intercept)    6.012     5.786    0.431   23.555 1.011      445      149
##
## Population-Level Effects:
##           Estimate Est.Error l-95% CI u-95% CI  Rhat Bulk_ESS Tail_ESS
## Intercept -106.501   114.054 -456.155   -5.931 1.013      368       97
## logsr       31.039    33.290    1.767  132.841 1.013      365       96
##
## Draws were sampled using sampling(NUTS). For each parameter, Bulk_ESS
## and Tail_ESS are effective sample size measures, and Rhat is the potential
## scale reduction factor on split chains (at convergence, Rhat = 1).
```

# Biological traits of exploited species

Bayesian multilevel regression models were used to compare the relative effects of four continuous species traits and four categorical funcional traits on predicting exploitation of individual species:

- `areakm2` = geographic range size in square kilometers
- `range_SST` = thermal range experienced by species across its geographic range in ºC
- `size` = shell size of species in mm
- `operational_bathymetry_min` = minimum bathymetric occurrence of species across its geographic range
- `operational_mobility` = whether a species is mobile or immobile
- `operational_fixation` = whether a species is attached or unattached to its substratum
- `operational_substrate` = substratum use of the species
- `operational_feeding` = feeding mode of the species.

The four continuous traits were z-standardized to better compare their effect sizes.

```
# rescale the variables for comparing effect sizes
trait_rescaled<-trait %>%
    mutate(areakm2=scale(log(areakm2)),
        range_SST=scale(range_SST),
        size=scale(log(size)),
        operational_bathymetry_min=scale(log(operational_bathymetry_min+1)))
```

We accounted for potential phylogenetic effects on the covariation of traits with exploitation by modeling a family-level offset (`(1|family)` below) and by the phylogenetic variance-covariance of the family-level phylogeny (`(1|gr(fam.tip, cov=A)` below). Certain families were operationally folded into those sampled on the phylogeny following Crouch et al. (2021, doi:10.1098/rspb.2021.2178), and see Supplemental Text.

We used default priors in the `brms` package. `A` is the variance-covariance matrix of the family phylogeny (`A <- ape::vcv.phylo(fam.tree)`, with the tip labels in variable `fam.tip`).

```
# model sampling
iter<-15000
chains<-5
cores<-5
warmup<-5000
thin<-1
```

```
seed<-2021

# without random effect
e_8trait_brm1<-brm(exploited ~ areakm2 + range_SST + size + operational_bathymetry_min +
    operational_feeding + operational_fixation + operational_mobility + operational_substrate,
                data=trait_rescaled,
                family=bernoulli(link="logit"),
                iter=iter, chains=chains, cores=cores, warmup=warmup, thin=thin,
                seed=seed)

# family identity as random effect
e_8trait_brm2<-brm(exploited ~ areakm2 + range_SST + size + operational_bathymetry_min +
    operational_feeding + operational_fixation + operational_mobility + operational_substrate +
                        (1|family),
                data=trait_rescaled,
                family=bernoulli(link="logit"),
                iter=iter, chains=chains, cores=cores, warmup=warmup, thin=thin,
                seed=seed)

# family phylogeny as the random effect
e_8trait_brm3<-brm(exploited ~ areakm2 + range_SST + size + operational_bathymetry_min +
    operational_feeding + operational_fixation + operational_mobility + operational_substrate+
                        (1|gr(fam.tip, cov=A)),
                data=trait_rescaled, data2=list(A=A),
                family=bernoulli(link="logit"),
                iter=iter, chains=chains, cores=cores, warmup=warmup, thin=thin,
                seed=seed)
```

We compared the three models in two ways: - using the expected log point-wise predictive density (ELPD) via leave-one-out cross-validation (loo), - and leave-one-group-out (logo, with `kfold`) cross-validation to account for the potential effects on estimating model performance by differently structured random effects (factor vs. variance-covariance matrix; see main text methods for details and references). We consider $\Delta$ELPD $>$ 2 to indicate significant improvement in model performance. In cases where $\Delta$ELPD $\leq 2$, we interpreted results with respect to the family-level intercept Model 2).

```
# LOO
e_8trait_brm_loo<-loo(e_8trait_brm1, e_8trait_brm2, e_8trait_brm3)

# LOGO
e_8trait_brm_kf2 <- kfold(e_8trait_brm2,compare=TRUE,K=NULL,
    Ksub=ids,folds=NULL,group='family',chains=1)

e_8trait_brm_kf3 <- kfold(e_8trait_brm3,compare=TRUE,K=NULL,
    Ksub=ids,folds=NULL,group='fam.tip',chains=1)

e_8trait_brm_logo<-loo_compare(e_8trait_brm_kf2,e_8trait_brm_kf3)
```

For each model (`mod`), we also calculated the area-under-the-curve (AUC) as a measure of prediction performance using `ROCit::rocit(score=predict(mod)[,1], class=y)$AUC`, with y representing the observed values:

```
# response variable for evaluating prediction performance
y<-trait_rescaled %>%
    filter(is.na(areakm2*range_SST*size*operational_bathymetry_min)==F) %>%
    pull(exploited)
```

Below, each model summary displays the specific model set-ups including the `Formula` at the top. The main parameters are under `Population-Level Effects`, including the intercept and the fixed effect. `sigma` in `Family Specific Parameters` represents the residual standard deviation of the response variable, and for Models 2 and 3, the `sd` in the `Group-Level Effects` indicates the standard deviation of the response variable under the random effect (here, membership in a taxonomic family as `family` or the effect of family phylogeny as `fam.tip`). We considered effects as significant if the 95% credible intervals (bracketed by the `l-95% CI` and `h-95% CI`) do not contain 0. All posterior samples are provided on the repository specified in the main text.

## Model comparison - LOO

Including the family identity improved the model performance and again, phylogeny does not significantly improve the model further.

```
mod_comp<-readRDS(paste(result_folder,"EIS_8trait_model_loo.rds", sep=""))
mod_comp
```

```
## Output of model 'e_8trait_brm1':
##
## Computed from 50000 by 5862 log-likelihood matrix
##
##          Estimate   SE
## elpd_loo  -1437.7 37.5
## p_loo        10.0  0.4
## looic      2875.4 75.0
## ------
## Monte Carlo SE of elpd_loo is 0.0.
##
## All Pareto k estimates are good (k < 0.5).
## See help('pareto-k-diagnostic') for details.
##
## Output of model 'e_8trait_brm2':
##
## Computed from 50000 by 5862 log-likelihood matrix
##
##          Estimate   SE
## elpd_loo  -1343.9 36.7
## p_loo        44.4  2.3
## looic      2687.7 73.3
## ------
## Monte Carlo SE of elpd_loo is 0.0.
##
## All Pareto k estimates are good (k < 0.5).
## See help('pareto-k-diagnostic') for details.
##
## Output of model 'e_8trait_brm3':
##
## Computed from 50000 by 5862 log-likelihood matrix
##
##          Estimate   SE
## elpd_loo  -1343.2 36.4
## p_loo        41.1  2.0
## looic      2686.3 72.9
## ------
## Monte Carlo SE of elpd_loo is 0.0.
```

```
##
## All Pareto k estimates are good (k < 0.5).
## See help('pareto-k-diagnostic') for details.
##
## Model comparisons:
##               elpd_diff se_diff
## e_8trait_brm3   0.0       0.0
## e_8trait_brm2  -0.7       2.6
## e_8trait_brm1 -94.5      13.6
```

### Model comparison - LOGO

Family phylogeny does not improve model performance when compared with family identity.

```
e_8trait_brm_kf2<-readRDS(paste(result_folder,"e_8trait_brm2_kf2.rds", sep=""))
e_8trait_brm_kf3<-readRDS(paste(result_folder,"e_8trait_brm2_kf3.rds", sep=""))
loo_compare(e_8trait_brm_kf2,e_8trait_brm_kf3)
```

```
##               elpd_diff se_diff
## e_8trait_brm2   0.0       0.0
## e_8trait_brm3 -17.1       2.8
```

### Model 1: traits

Several functional traits affected the species' likelihood of being exploited in addition to the continuous traits.

```
mod<-readRDS(paste(result_folder,"EIS_8trait_nofamnophylo.rds", sep=""))
mod.predict<-predict(mod)
mod.auc<-ROCit::rocit(score=mod.predict[,1], class=y)$AUC
mod
```

```
##  Family: bernoulli
##   Links: mu = logit
## Formula: exploited ~ areakm2 + range_SST + size + operational_bathymetry_min + operational_feeding +
##    Data: trait_rescaled (Number of observations: 5862)
##   Draws: 5 chains, each with iter = 15000; warmup = 5000; thin = 1;
##          total post-warmup draws = 50000
##
## Population-Level Effects:
##                                    Estimate Est.Error l-95% CI u-95% CI Rhat
## Intercept                             -5.18      0.27    -5.72    -4.66 1.00
## areakm2                                0.45      0.10     0.26     0.65 1.00
## range_SST                              0.30      0.06     0.18     0.42 1.00
## size                                   2.28      0.10     2.09     2.47 1.00
## operational_bathymetry_min            -0.91      0.07    -1.05    -0.76 1.00
## operational_feedingnon_suspension      0.22      0.22    -0.21     0.65 1.00
## operational_feedingsuspension          0.82      0.19     0.45     1.20 1.00
## operational_fixationunattached         0.36      0.18     0.01     0.71 1.00
## operational_mobilitymobile             0.59      0.16     0.28     0.90 1.00
## operational_substrateinfaunal         -0.09      0.17    -0.42     0.24 1.00
##                                    Bulk_ESS Tail_ESS
## Intercept                             42319    38479
## areakm2                               49656    38640
## range_SST                             50890    40840
## size                                  43890    37809
## operational_bathymetry_min            57318    38306
```

```
## operational_feedingnon_suspension    44501    37747
## operational_feedingsuspension        44112    37376
## operational_fixationunattached       46075    37830
## operational_mobilitymobile           55507    38048
## operational_substrateinfaunal        52937    38285
##
## Draws were sampled using sampling(NUTS). For each parameter, Bulk_ESS
## and Tail_ESS are effective sample size measures, and Rhat is the potential
## scale reduction factor on split chains (at convergence, Rhat = 1).
```

AUC for this model is 0.908.

### Model 2: traits+family

Family significantly effects the presence of exploited species, but most of the functional effects are absent in this model.

```
mod<-readRDS(paste(result_folder,"EIS_8trait_family.rds", sep=""))
mod.predict<-predict(mod)
mod.auc<-ROCit::rocit(score=mod.predict[,1], class=y)$AUC
mod
```

```
##  Family: bernoulli
##   Links: mu = logit
## Formula: exploited ~ areakm2 + range_SST + size + operational_bathymetry_min + operational_feeding +
##    Data: trait_rescaled (Number of observations: 5862)
##   Draws: 5 chains, each with iter = 15000; warmup = 5000; thin = 1;
##          total post-warmup draws = 50000
##
## Group-Level Effects:
## ~family (Number of levels: 80)
##               Estimate Est.Error l-95% CI u-95% CI Rhat Bulk_ESS Tail_ESS
## sd(Intercept)     1.10      0.17     0.81     1.47 1.00    10559    19534
##
## Population-Level Effects:
##                                    Estimate Est.Error l-95% CI u-95% CI Rhat
## Intercept                             -5.58      0.93    -7.38    -3.71 1.00
## areakm2                                0.55      0.11     0.35     0.77 1.00
## range_SST                              0.33      0.07     0.20     0.46 1.00
## size                                   2.66      0.12     2.43     2.90 1.00
## operational_bathymetry_min            -0.73      0.08    -0.89    -0.58 1.00
## operational_feedingnon_suspension     -0.30      0.93    -2.19     1.48 1.00
## operational_feedingsuspension          0.34      0.89    -1.47     2.03 1.00
## operational_fixationunattached         0.68      0.28     0.14     1.23 1.00
## operational_mobilitymobile             0.34      0.21    -0.08     0.76 1.00
## operational_substrateinfaunal         -0.12      0.24    -0.58     0.35 1.00
##                                    Bulk_ESS Tail_ESS
## Intercept                             17074    24141
## areakm2                               36430    36619
## range_SST                             38777    37583
## size                                  35517    35121
## operational_bathymetry_min            48539    39026
## operational_feedingnon_suspension     16992    23727
## operational_feedingsuspension         17190    23156
## operational_fixationunattached        23668    32735
```

```
## operational_mobilitymobile           30697    35891
## operational_substrateinfaunal        29222    35245
##
## Draws were sampled using sampling(NUTS). For each parameter, Bulk_ESS
## and Tail_ESS are effective sample size measures, and Rhat is the potential
## scale reduction factor on split chains (at convergence, Rhat = 1).
```

AUC for this model is 0.927.

## Model 3: traits+phylogeny

Family phylogeny significantly effects the presence of exploited species.

```
mod<-readRDS(paste(result_folder,"EIS_8trait_phylo.rds", sep=""))
mod.predict<-predict(mod)
mod.auc<-ROCit::rocit(score=mod.predict[,1], class=y)$AUC
mod
```

```
##  Family: bernoulli
##   Links: mu = logit
## Formula: exploited ~ areakm2 + range_SST + size + operational_bathymetry_min + operational_feeding +
##    Data: trait_rescaled (Number of observations: 5862)
##   Draws: 5 chains, each with iter = 15000; warmup = 5000; thin = 1;
##          total post-warmup draws = 50000
##
## Group-Level Effects:
## ~fam.tip (Number of levels: 80)
##               Estimate Est.Error l-95% CI u-95% CI Rhat Bulk_ESS Tail_ESS
## sd(Intercept)     0.06      0.01     0.04     0.08 1.00    17095    28883
##
## Population-Level Effects:
##                                     Estimate Est.Error l-95% CI u-95% CI Rhat
## Intercept                              -6.32      0.78    -7.87    -4.81 1.00
## areakm2                                 0.54      0.11     0.34     0.75 1.00
## range_SST                               0.32      0.07     0.19     0.45 1.00
## size                                    2.64      0.12     2.41     2.88 1.00
## operational_bathymetry_min             -0.73      0.08    -0.89    -0.57 1.00
## operational_feedingnon_suspension       0.15      0.64    -1.13     1.39 1.00
## operational_feedingsuspension           0.97      0.64    -0.30     2.20 1.00
## operational_fixationunattached          0.61      0.27     0.08     1.16 1.00
## operational_mobilitymobile              0.37      0.21    -0.05     0.79 1.00
## operational_substrateinfaunal          -0.19      0.24    -0.67     0.29 1.00
##                                     Bulk_ESS Tail_ESS
## Intercept                              27852    32809
## areakm2                                74564    42315
## range_SST                              77312    41962
## size                                   85849    37214
## operational_bathymetry_min            107416    37487
## operational_feedingnon_suspension      32941    33566
## operational_feedingsuspension          32848    32512
## operational_fixationunattached         58173    40955
## operational_mobilitymobile             60602    41092
## operational_substrateinfaunal          69043    41047
##
## Draws were sampled using sampling(NUTS). For each parameter, Bulk_ESS
```

```
## and Tail_ESS are effective sample size measures, and Rhat is the potential
## scale reduction factor on split chains (at convergence, Rhat = 1).
```

AUC for this model is 0.926

## Trait distributions

Class-wide distributions of exploited and non-exploited species across the four continuous traits (shell size, minimum bathymetry, geographic range size, and thermal range) were compared using Kolmogorov-Smirnov tests.

```
ks.result<-list()

# size
x1<-trait %>% filter(exploited==TRUE) %>% pull(size)
x2<-trait %>% filter(exploited==FALSE) %>% pull(size)
ks.result[['size']]<-ks.test(log(x1), log(x2), alternative="less") # x1>x2

# geographic range
x1<-trait %>% filter(exploited==TRUE) %>% pull(areakm2)
x2<-trait %>% filter(exploited==FALSE) %>% pull(areakm2)
ks.result[['areakm2']]<-ks.test(log(x1), log(x2), alternative="less") # x1>x2

# thermal range
x1<-trait %>% filter(exploited==TRUE) %>% pull(range_SST)
x2<-trait %>% filter(exploited==FALSE) %>% pull(range_SST)
ks.result[['range_SST']]<-ks.test(x1, x2, alternative="less") # x1>x2

# minimum bathymetry
x1<-trait %>% filter(exploited==TRUE) %>% pull(operational_bathymetry_min)
x2<-trait %>% filter(exploited==FALSE) %>% pull(operational_bathymetry_min)
ks.result[['operational_bathymetry_min']]<-ks.test(log(x1+1), log(x2+1), alternative="great") # x1<x2

# print result
ks.result
```

```
## $size
##
##  Asymptotic two-sample Kolmogorov-Smirnov test
##
## data:  log(x1) and log(x2)
## D^- = 0.61448, p-value < 2.2e-16
## alternative hypothesis: the CDF of x lies below that of y
##
##
## $areakm2
##
##  Asymptotic two-sample Kolmogorov-Smirnov test
##
## data:  log(x1) and log(x2)
## D^- = 0.31204, p-value < 2.2e-16
## alternative hypothesis: the CDF of x lies below that of y
##
##
## $range_SST
```

```
##
##  Asymptotic two-sample Kolmogorov-Smirnov test
##
## data:  x1 and x2
## D^- = 0.29008, p-value < 2.2e-16
## alternative hypothesis: the CDF of x lies below that of y
##
##
## $operational_bathymetry_min
##
##  Asymptotic two-sample Kolmogorov-Smirnov test
##
## data:  log(x1 + 1) and log(x2 + 1)
## D^+ = 0.40369, p-value < 2.2e-16
## alternative hypothesis: the CDF of x lies above that of y
```

# Intrinsic vulnerability of exploited species

## Family extinction history

The per-genus extinction rate of families through the Cenozoic (`qhatH`) was not log-transformed.

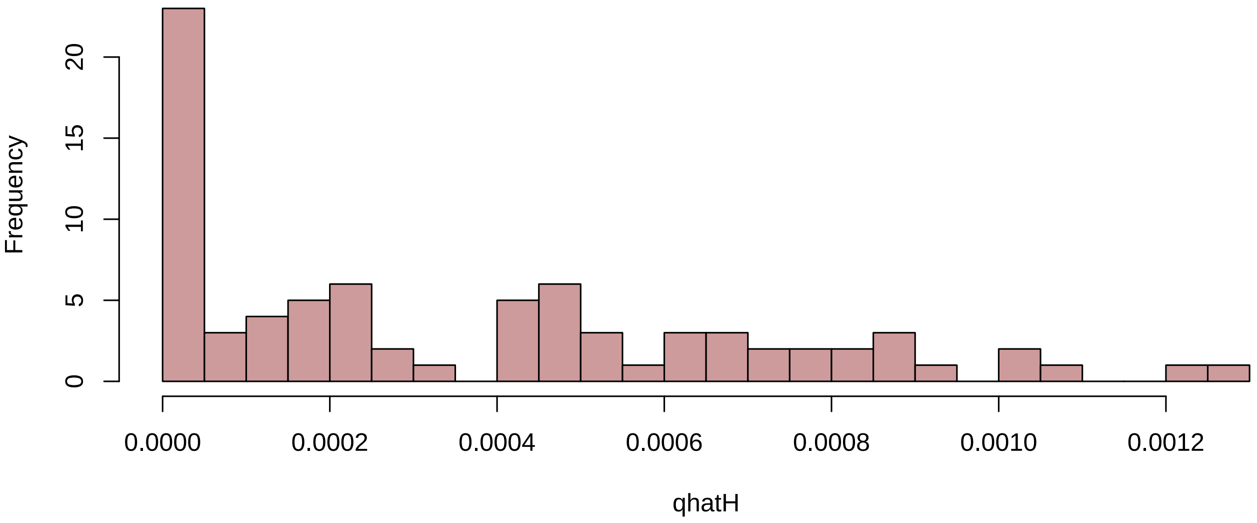


To compare family extinction histories, we quantified exploitation at the family level as a binary variable of whether a family has any exploited species. We comapred a simpler model with only the fixed effect (`fam_q_brm1.1`) and one phylogenetically informed model using the variance-covariance matrix from the family-level phylogeny as the random effect (`fam_q_brm1.2`).

```
# simple model without phylogeny
fam_q_brm1.1<-brm(exploited_bin ~ qhatH,
    data=fam.eis,
    family=bernoulli(link="logit"),
    # control=list(adapt_delta=0.9),
    iter=iter, chains=chains, cores=cores, warmup=warmup, thin=thin, seed=seed)

# phylogenetically infomred model
fam_q_brm1.2<-brm(exploited_bin ~ qhatH + (1|gr(family, cov=A)),
    data=fam.eis, data2=list(A=A),
    family=bernoulli(link="logit"),
```

```
    # control=list(adapt_delta=0.9),
    iter=iter, chains=chains, cores=cores, warmup=warmup, thin=thin, seed=seed)
```

#### Model comparison

We compared the simple and phylogenetically informed model using the expected log point-wise predictive density (ELPD) via leave-one-out cross-validation (loo). Including the phylogenetic information significantly improved model performance, but resulted in low convergence rate (based on the Pareto k diagnostic values).

```
mod_comp<-readRDS(paste(result_folder,"qhat_EIS_model_loo.rds", sep=""))
mod_comp[[1]]
```

```
## Output of model 'fam_q_brm1.1':
##
## Computed from 50000 by 80 log-likelihood matrix
##
##          Estimate  SE
## elpd_loo    -57.2 1.1
## p_loo         2.2 0.2
## looic       114.3 2.2
## ------
## Monte Carlo SE of elpd_loo is 0.0.
##
## All Pareto k estimates are good (k < 0.5).
## See help('pareto-k-diagnostic') for details.
##
## Output of model 'fam_q_brm1.2':
##
## Computed from 50000 by 80 log-likelihood matrix
##
##          Estimate  SE
## elpd_loo    -11.4 1.1
## p_loo         9.7 1.0
## looic        22.9 2.3
## ------
## Monte Carlo SE of elpd_loo is NA.
##
## Pareto k diagnostic values:
##                          Count Pct.    Min. n_eff
## (-Inf, 0.5]   (good)      0     0.0%   <NA>
##  (0.5, 0.7]   (ok)        0     0.0%   <NA>
##    (0.7, 1]   (bad)      62    77.5%   96
##    (1, Inf)   (very bad) 18    22.5%   15
## See help('pareto-k-diagnostic') for details.
##
## Model comparisons:
##              elpd_diff se_diff
## fam_q_brm1.2   0.0       0.0
## fam_q_brm1.1 -45.7       1.4
```

**Individual-level model** Exploited and unexploited families experienced similar genus-level extinction rates (`qhatH`) through the Cenozoic.

```r
mod<-readRDS(paste(result_folder,"fam_qhat_EISbin.rds", sep=""))
mod
```

```
##  Family: bernoulli
##   Links: mu = logit
## Formula: exploited_bin ~ qhatH
##    Data: fam.eis (Number of observations: 80)
##   Draws: 5 chains, each with iter = 15000; warmup = 5000; thin = 1;
##          total post-warmup draws = 50000
##
## Population-Level Effects:
##           Estimate Est.Error l-95% CI u-95% CI Rhat Bulk_ESS Tail_ESS
## Intercept     0.12      0.33    -0.52     0.77 1.00    43610    31641
## qhatH       235.58    674.34 -1078.68  1581.88 1.00    41933    32257
##
## Draws were sampled using sampling(NUTS). For each parameter, Bulk_ESS
## and Tail_ESS are effective sample size measures, and Rhat is the potential
## scale reduction factor on split chains (at convergence, Rhat = 1).
```

### Phylogenetically informed model

Family phylogeny significantly effects the correlation between extinction rate and exploitation.

```r
mod<-readRDS(paste(result_folder,"fam_qhat_EISbin_phylo.rds", sep=""))
mod
```

```
##  Family: bernoulli
##   Links: mu = logit
## Formula: exploited_bin ~ qhatH + (1 | gr(family, cov = A))
##    Data: fam.eis (Number of observations: 80)
##   Draws: 5 chains, each with iter = 15000; warmup = 5000; thin = 1;
##          total post-warmup draws = 50000
##
## Group-Level Effects:
## ~family (Number of levels: 80)
##               Estimate Est.Error l-95% CI u-95% CI Rhat Bulk_ESS Tail_ESS
## sd(Intercept)     6.67      7.73     0.51    29.73 1.01      499      152
##
## Population-Level Effects:
##           Estimate Est.Error   l-95% CI u-95% CI Rhat Bulk_ESS Tail_ESS
## Intercept     1.10     20.97     -35.57    54.50 1.01      350      104
## qhatH     -3367.61  57530.77 -151025.33 96006.97 1.01      352      104
##
## Draws were sampled using sampling(NUTS). For each parameter, Bulk_ESS
## and Tail_ESS are effective sample size measures, and Rhat is the potential
## scale reduction factor on split chains (at convergence, Rhat = 1).
```

## PERIL score

The species PERIL scores (`comboPERILfull`) of bivalve species are normally distributed, and therefore not log transformed for analysis.

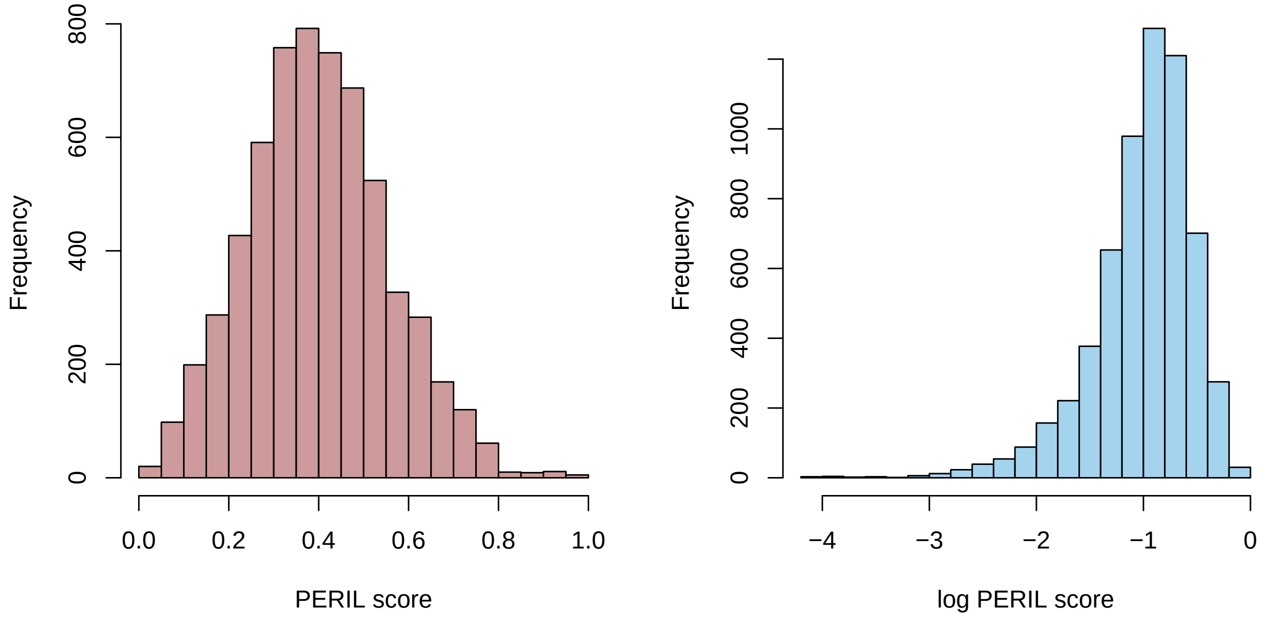


Similar to the model using species traits, we accounted for potential phylogenetic effects on the covariation of traits with exploitation by modeling a family-level offset (`(1|family)` below) and by the phylogenetic variance-covariance of the family-level phylogeny (`(1|gr(fam.tip, cov=A)` below). In addition, we tested a fourth model where the PERIL effect is allowed to vary by family, to test whether exploitation probability consistently decreases with increasing PERIL score across families.

```
# without random effect
e_peril_brm1<-brm(exploited ~ comboPERILfull,
                data=trait,
                family=bernoulli(link="logit"),
                iter=iter, chains=chains, cores=cores, warmup=warmup, thin=thin,
                seed=seed)

# family identity as random effect
e_peril_brm2<-brm(exploited ~ comboPERILfull + (1|family),
                data=trait,
                family=bernoulli(link="logit"),
                iter=iter, chains=chains, cores=cores, warmup=warmup, thin=thin,
                seed=seed)

# family phylogeny as the random effect
e_peril_brm3<-brm(exploited ~ comboPERILfull + (1|gr(fam.tip, cov=A)),
                data=trait, data2=list(A=A),
                family=bernoulli(link="logit"),
                iter=iter, chains=chains, cores=cores, warmup=warmup, thin=thin,
                seed=seed)

# family identity as the random effect on the slope
e_peril_brm_4<-brm(comboPERILfull ~ exploited + (exploited|family),
                data=trait, family=gaussian(),
                iter=iter, chains=chains, cores=cores, warmup=warmup, thin=thin,
                seed=seed)
```

```r
# model comparison
e_peril_brm_loo<-loo(e_peril_brm_1, e_peril_brm_2, e_peril_brm_3, e_peril_brm_4)
```

### Model comparison

We compared the simple and phylogenetically informed model using the expected log point-wise predictive density (ELPD) via leave-one-out cross-validation (loo). Including the family identity improved the model performance, which is similar to that of the model including the family phylogeny (`peril_brm_3`).

```r
mod_comp<-readRDS(paste(result_folder,"EIS_PERIL_model_loo.rds", sep=""))
mod_comp
```

```
## Output of model 'e_peril_brm_1':
##
## Computed from 50000 by 6127 log-likelihood matrix
##
##          Estimate   SE
## elpd_loo  -2284.5 49.0
## p_loo         1.9  0.1
## looic      4569.1 97.9
## ------
## Monte Carlo SE of elpd_loo is 0.0.
##
## All Pareto k estimates are good (k < 0.5).
## See help('pareto-k-diagnostic') for details.
##
## Output of model 'e_peril_brm_2':
##
## Computed from 50000 by 6127 log-likelihood matrix
##
##          Estimate   SE
## elpd_loo  -1843.7 42.5
## p_loo        48.7  3.8
## looic      3687.3 85.1
## ------
## Monte Carlo SE of elpd_loo is 0.1.
##
## Pareto k diagnostic values:
##                          Count Pct.    Min. n_eff
## (-Inf, 0.5]   (good)     6120  99.9%   3805
##  (0.5, 0.7]   (ok)          7   0.1%   1633
##    (0.7, 1]   (bad)         0   0.0%   <NA>
##    (1, Inf)   (very bad)    0   0.0%   <NA>
##
## All Pareto k estimates are ok (k < 0.7).
## See help('pareto-k-diagnostic') for details.
##
## Output of model 'e_peril_brm_3':
##
## Computed from 50000 by 6127 log-likelihood matrix
##
##          Estimate   SE
## elpd_loo  -1845.3 42.5
## p_loo        45.8  3.4
```

```
## looic      3690.5 85.0
## ------
## Monte Carlo SE of elpd_loo is 0.0.
##
## Pareto k diagnostic values:
##                          Count Pct.    Min. n_eff
## (-Inf, 0.5]   (good)     6124  100.0%  9740
##  (0.5, 0.7]   (ok)          3    0.0%  5105
##    (0.7, 1]   (bad)         0    0.0%  <NA>
##    (1, Inf)   (very bad)    0    0.0%  <NA>
##
## All Pareto k estimates are ok (k < 0.7).
## See help('pareto-k-diagnostic') for details.
##
## Output of model 'e_peril_brm_4':
##
## Computed from 50000 by 6127 log-likelihood matrix
##
##          Estimate   SE
## elpd_loo  -1843.4 42.5
## p_loo        52.2  3.9
## looic      3686.9 85.0
## ------
## Monte Carlo SE of elpd_loo is NA.
##
## Pareto k diagnostic values:
##                          Count Pct.    Min. n_eff
## (-Inf, 0.5]   (good)     6117  99.8%   8471
##  (0.5, 0.7]   (ok)          9   0.1%   2607
##    (0.7, 1]   (bad)         1   0.0%   1079
##    (1, Inf)   (very bad)    0   0.0%   <NA>
## See help('pareto-k-diagnostic') for details.
##
## Model comparisons:
##               elpd_diff se_diff
## e_peril_brm_4    0.0       0.0
## e_peril_brm_2   -0.2       1.0
## e_peril_brm_3   -1.8       2.5
## e_peril_brm_1 -441.1      26.0
```

### Model 1: PERIL

Without considering any family effect (either family identity or the family phylogeny), exploited species with lower PERIL scores are more likely to be exploited than those with higher PERIL scores.

```
mod<-readRDS(paste(result_folder,"EIS_PERIL_nofamnophylo.rds", sep=""))
mod
```

```
##  Family: bernoulli
##   Links: mu = logit
## Formula: exploited ~ comboPERILfull
##    Data: trait (Number of observations: 6127)
##   Draws: 5 chains, each with iter = 15000; warmup = 5000; thin = 1;
##          total post-warmup draws = 50000
##
```

```
## Population-Level Effects:
##                 Estimate Est.Error l-95% CI u-95% CI Rhat Bulk_ESS Tail_ESS
## Intercept          -0.59      0.10    -0.79    -0.39 1.00    38626    34624
## comboPERILfull     -3.53      0.27    -4.07    -3.00 1.00    29114    30247
##
## Draws were sampled using sampling(NUTS). For each parameter, Bulk_ESS
## and Tail_ESS are effective sample size measures, and Rhat is the potential
## scale reduction factor on split chains (at convergence, Rhat = 1).
```

### Model 2: PERIL+family

Family identity significantly effects species' PERIL scores, and exploited species tend to have lower PERIL scores than non-exploited species within families.

```
mod<-readRDS(paste(result_folder,"EIS_PERIL_family.rds", sep=""))
mod
```

```
##  Family: bernoulli
##   Links: mu = logit
## Formula: exploited ~ comboPERILfull + (1 | family)
##    Data: trait (Number of observations: 6127)
##   Draws: 5 chains, each with iter = 15000; warmup = 5000; thin = 1;
##          total post-warmup draws = 50000
##
## Group-Level Effects:
## ~family (Number of levels: 80)
##               Estimate Est.Error l-95% CI u-95% CI Rhat Bulk_ESS Tail_ESS
## sd(Intercept)     2.31      0.31     1.78     2.99 1.00     7595    13814
##
## Population-Level Effects:
##                 Estimate Est.Error l-95% CI u-95% CI Rhat Bulk_ESS Tail_ESS
## Intercept          -1.00      0.36    -1.73    -0.34 1.00     5839    11327
## comboPERILfull     -6.32      0.38    -7.08    -5.57 1.00    45010    37347
##
## Draws were sampled using sampling(NUTS). For each parameter, Bulk_ESS
## and Tail_ESS are effective sample size measures, and Rhat is the potential
## scale reduction factor on split chains (at convergence, Rhat = 1).
```

### Model 3: PERIL+phylogeny

Family phylogeny significantly effects species' PERIL scores, and exploited species tend to have lower PERIL scores than non-exploited species within families.

```
mod<-readRDS(paste(result_folder,"EIS_PERIL_phylo.rds", sep=""))
mod
```

```
##  Family: bernoulli
##   Links: mu = logit
## Formula: exploited ~ comboPERILfull + (1 | gr(fam.tip, cov = A))
##    Data: trait (Number of observations: 6127)
##   Draws: 5 chains, each with iter = 15000; warmup = 5000; thin = 1;
##          total post-warmup draws = 50000
##
## Group-Level Effects:
## ~fam.tip (Number of levels: 80)
##               Estimate Est.Error l-95% CI u-95% CI Rhat Bulk_ESS Tail_ESS
```

```
## sd(Intercept)     0.12      0.02     0.09     0.16 1.00    13815    25582
##
## Population-Level Effects:
##                Estimate Est.Error l-95% CI u-95% CI Rhat Bulk_ESS Tail_ESS
## Intercept         -1.49      0.73    -2.99    -0.12 1.00    12035    20682
## comboPERILfull    -6.27      0.38    -7.02    -5.53 1.00   104282    37527
##
## Draws were sampled using sampling(NUTS). For each parameter, Bulk_ESS
## and Tail_ESS are effective sample size measures, and Rhat is the potential
## scale reduction factor on split chains (at convergence, Rhat = 1).
```

### Model 4: family-specific effect

Families tend to have similar differences in PERIL scores between exploited and non-exploited species, and overall, exploited species have lower PERIL scores than non-exploited species.

```
mod<-readRDS(paste(result_folder,"EIS_PERIL_family_on_slope.rds", sep=""))
mod
```

```
##  Family: bernoulli
##   Links: mu = logit
## Formula: exploited ~ comboPERILfull + (comboPERILfull | family)
##    Data: trait (Number of observations: 6127)
##   Draws: 5 chains, each with iter = 15000; warmup = 5000; thin = 1;
##          total post-warmup draws = 50000
##
## Group-Level Effects:
## ~family (Number of levels: 80)
##                               Estimate Est.Error l-95% CI u-95% CI Rhat
## sd(Intercept)                     2.15      0.36     1.51     2.92 1.00
## sd(comboPERILfull)                1.09      0.69     0.06     2.62 1.00
## cor(Intercept,comboPERILfull)     0.35      0.49    -0.76     0.98 1.00
##                               Bulk_ESS Tail_ESS
## sd(Intercept)                    10714    18211
## sd(comboPERILfull)                5803     9270
## cor(Intercept,comboPERILfull)    23797    24449
##
## Population-Level Effects:
##                Estimate Est.Error l-95% CI u-95% CI Rhat Bulk_ESS Tail_ESS
## Intercept         -0.85      0.37    -1.63    -0.17 1.00     6659    10623
## comboPERILfull    -6.88      0.72    -8.48    -5.62 1.00    13103    11273
##
## Draws were sampled using sampling(NUTS). For each parameter, Bulk_ESS
## and Tail_ESS are effective sample size measures, and Rhat is the potential
## scale reduction factor on split chains (at convergence, Rhat = 1).
```

### PERIL distributions

We also compared the distributions of exploited and non-exploited species on PERIL scores using the Kolmogorov-Smirnov test.

```
x1<-trait %>% filter(exploited==TRUE) %>% pull(comboPERILfull)
x2<-trait %>% filter(exploited==FALSE) %>% pull(comboPERILfull)
ks.test(x1, x2, alternative="greater") # x1<x2
```

```
##
```

```
##  Asymptotic two-sample Kolmogorov-Smirnov test
##
## data:  x1 and x2
## D^+ = 0.20373, p-value < 2.2e-16
## alternative hypothesis: the CDF of x lies above that of y
```

# Biogeography of exploited species

Spatial variation in extinction risk of exploited species was analyzed as both the number and proportion of exploited species in a grid cell with PERIL scores above the global median (globally vulnerable species) or above the median within a region, i.e. the intersection of coastlines with polar, temperate, and tropical climate zones (regionally vulnerable species). We further identified highly vulnerable species as those with PERIL scores in the top 20% or 10% globally or regionally.

```
# global PERIL thresholds
pmed_global<-median(sp.trait$comboPERILfull, na.rm=T)
p80_global<-quantile(sp.trait$comboPERILfull, 0.8, na.rm=T)
p90_global<-quantile(sp.trait$comboPERILfull, 0.9, na.rm=T)

# PERIL threholds by climate-coast region
spp.cc<-unique(spp.occ[c("valid_species_id", "peril", "ClimCoast")])
cc<-spp.cc %>%
            select(ClimCoast, sr_cc=valid_species_id, pmed_cc=peril) %>%
            group_by(ClimCoast) %>%
            mutate(
                p90_cc=quantile(pmed_cc, prob=0.9, na.rm=T),
                p80_cc=quantile(pmed_cc, prob=0.8, na.rm=T),
                pmed_cc=median(pmed_cc, na.rm=T),
                sr_cc=length(unique(sr_cc))
            ) %>%
            unique()
spp.occ<-left_join(spp.occ, cc, by="ClimCoast")
```

Spatial patterns of exploited and non-exploited species richness, and their intrinsic vulnerability (based on the PERIL scores), were quantified using their occurrences on the global 50x50 km grid intersected with the continental shelf.

```
# summarised by grid
sr.grid<-spp.occ %>%
            select(EAGid, sr=valid_species_id, sr_cc, exploited, fao.prod,
                peril, climate, coastline, pmed_cc, p90_cc, p80_cc) %>%
            group_by(EAGid) %>%
            mutate(
                global_high_peril_eis=sum(exploited[which(peril > pmed_global)]),
                global_high80_peril_eis=sum(exploited[which(peril > p80_global)]),
                global_high90_peril_eis=sum(exploited[which(peril > p90_global)]),
                cc_high_peril_eis=sum(exploited[which(peril>pmed_cc)]),
                cc_high90_peril_eis=sum(exploited[which(peril>p90_cc)]),
                cc_high80_peril_eis=sum(exploited[which(peril>p80_cc)]),
                global_high_peril=length(which(peril > pmed_global)),
                global_high80_peril=length(which(peril > p80_global)),
                global_high90_peril=length(which(peril > p90_global)),
                cc_high_peril=length(which(peril>pmed_cc)),
                cc_high90_peril=length(which(peril>p90_cc)),
                cc_high80_peril=length(which(peril>p80_cc)),
```

```
    cc_low_peril=length(which(peril<=pmed_cc)),
    sr=length(unique(sr)),
    exploited=sum(exploited),
    fao=sum(fao.prod),
    peril=mean(peril, na.rm=T)) %>%
distinct()
```
