# Supplementary material for: Diversity, distribution and intrinsic extinction vulnerability of exploited marine bivalves
Source: Nat Commun. 2023 Aug 15;14:4639. doi: 10.1038/s41467-023-40053-y (PMC10427664; doi:10.1038/s41467-023-40053-y)
Supplement: Supplementary file 6 — Reporting Summary [file 41467_2023_40053_MOESM6_ESM.pdf]

nature portfolio

Corresponding author(s): *Double-anonymous peer review submissions: write DAPR and your manuscript number here instead of author names.*

Last updated by author(s): Jun 15, 2023

Reporting Summary

Nature Portfolio wishes to improve the reproducibility of the work that we publish. This form provides structure for consistency and transparency in reporting. For further information on Nature Portfolio policies, see our Editorial Policies and the Editorial Policy Checklist.

Statistics

For all statistical analyses, confirm that the following items are present in the figure legend, table legend, main text, or Methods section.

n/a	Confirmed	
☐	☒	The exact sample size (*n*) for each experimental group/condition, given as a discrete number and unit of measurement
☐	☒	A statement on whether measurements were taken from distinct samples or whether the same sample was measured repeatedly
☐	☒	The statistical test(s) used AND whether they are one- or two-sided<br>*Only common tests should be described solely by name; describe more complex techniques in the Methods section.*
☐	☒	A description of all covariates tested
☐	☒	A description of any assumptions or corrections, such as tests of normality and adjustment for multiple comparisons
☐	☒	A full description of the statistical parameters including central tendency (e.g. means) or other basic estimates (e.g. regression coefficient) AND variation (e.g. standard deviation) or associated estimates of uncertainty (e.g. confidence intervals)
☐	☒	For null hypothesis testing, the test statistic (e.g. *F*, *t*, *r*) with confidence intervals, effect sizes, degrees of freedom and *P* value noted<br>*Give P values as exact values whenever suitable.*
☐	☒	For Bayesian analysis, information on the choice of priors and Markov chain Monte Carlo settings
☐	☒	For hierarchical and complex designs, identification of the appropriate level for tests and full reporting of outcomes
☐	☒	Estimates of effect sizes (e.g. Cohen's *d*, Pearson's *r*), indicating how they were calculated

Our web collection on statistics for biologists contains articles on many of the points above.

Software and code

Policy information about availability of computer code

Data collection	We did not use any particular software for data collection.
Data analysis	All data were processed and analyzed in R 4.2.2 with the packages 'tidyverse', 'Hmisc' and 'ggtree' for dataset visualization and transformation, 'sf' for spatial analyses, 'ape', 'phytools'and 'caper' for phylogenetic analyses, and 'brms'and 'loo' for Bayesian multilevel regression analyses and model comparison. All analytical code is provided in Appendix 1. The code for producing all figures and tables is diposited on FigShare (https://doi.org/10.6084/m9.figshare.22696279).

For manuscripts utilizing custom algorithms or software that are central to the research but not yet described in published literature, software must be made available to editors and reviewers. We strongly encourage code deposition in a community repository (e.g. GitHub). See the Nature Portfolio guidelines for submitting code & software for further information.

Data

Policy information about availability of data

All manuscripts must include a data availability statement. This statement should provide the following information, where applicable:

- Accession codes, unique identifiers, or web links for publicly available datasets
- A description of any restrictions on data availability
- For clinical datasets or third party data, please ensure that the statement adheres to our policy

All data used in this study are provided in Supplementary Information, Dataset S1 and S2. The posterior samples from Bayesian models generated in this study and the shapefile of the global equal-area grids are deposited on FigShare (https://doi.org/10.6084/m9.figshare.22696279).

Research involving human participants, their data, or biological material

Policy information about studies with human participants or human data. See also policy information about sex, gender (identity/presentation), and sexual orientation and race, ethnicity and racism.

Reporting on sex and gender	N/A
Reporting on race, ethnicity, or other socially relevant groupings	N/A
Population characteristics	N/A
Recruitment	N/A
Ethics oversight	N/A

Note that full information on the approval of the study protocol must also be provided in the manuscript.

Field-specific reporting

Please select the one below that is the best fit for your research. If you are not sure, read the appropriate sections before making your selection.

☐ Life sciences ☐ Behavioural & social sciences ☒ Ecological, evolutionary & environmental sciences

For a reference copy of the document with all sections, see nature.com/documents/nr-reporting-summary-flat.pdf

Ecological, evolutionary & environmental sciences study design

All studies must disclose on these points even when the disclosure is negative.

Study description	We analyzed the phylogenetic distributions, biological traits and global biogeography of exploited species in shallow-marine bivalves to assess their intrinsic vulnerability to ongoing and future global change. Exploitation was considered as a binary variable. Four traits are continuous variables: body size, geographic range size, minimum bathymetry and thermal range. Body size and geographic range size were log-transformed to increase normality. Four additional traits are categorical variables: feeding type, living position, substratum attachment, and mobility. Traits between exploited and non-exploited species were compared using hierarchical logistic regressions with additive effects of all eight traits and a random effect of the family identity or family phylogeny (reconstructed in a previous study by Crouch et al. 2021). No interaction term was included. Global occurrence is binary, as presence/abscence in each 50x50 km grid.
Research sample	We studied all shallow-marine bivalves species with occurrence and trait data available from an actively maintained and taxonomically standardized database. We also compiled a list of any exploited species reported in the literature. Our data are meant to represent the global shallow-marine bivalves living today.
Sampling strategy	We included all species with complete data for the focal traits and no sampling procedure was involved.
Data collection	All data were downloaded from an actively maintained and taxonomically standardized database (curated by the authors of the paper).
Timing and spatial scale	We conducted a literature survey without a predefined time window of publication and included records of all shallow-marine bivalve across the world.
Data exclusions	No data were excluded.
Reproducibility	No experiment was invovled. All analytical code and data are provided for reproducing the results.

Randomization	Exploited species were identified based on the literature survey and any significant difference between exploited and non-exploited species is tested against random distributions.
Blinding	We included any marine bivalve species that have been reported as food source in the literature. The data were derived from an open search of the primary literature and thus blinding was not necessary.

Did the study involve field work? ☐ Yes ☒ No

Reporting for specific materials, systems and methods

We require information from authors about some types of materials, experimental systems and methods used in many studies. Here, indicate whether each material, system or method listed is relevant to your study. If you are not sure if a list item applies to your research, read the appropriate section before selecting a response.

Materials & experimental systems

n/a	Involved in the study
☒	☐ Antibodies
☒	☐ Eukaryotic cell lines
☒	☐ Palaeontology and archaeology
☒	☐ Animals and other organisms
☒	☐ Clinical data
☒	☐ Dual use research of concern
☒	☐ Plants

Methods

n/a	Involved in the study
☒	☐ ChIP-seq
☒	☐ Flow cytometry
☒	☐ MRI-based neuroimaging
